# CAR T cells with dual targeting of CD19 and CD22 in pediatric and young adult patients with relapsed or refractory B cell acute lymphoblastic leukemia: a phase 1 trial

**DOI:** 10.1038/s41591-021-01497-1

**Published:** 2021-10-12

**Authors:** Shaun Cordoba, Shimobi Onuoha, Simon Thomas, Daniela Soriano Pignataro, Rachael Hough, Sara Ghorashian, Ajay Vora, Denise Bonney, Paul Veys, Kanchan Rao, Giovanna Lucchini, Robert Chiesa, Jan Chu, Liz Clark, Mei Mei Fung, Koval Smith, Carlotta Peticone, Muhammad Al-Hajj, Vania Baldan, Mathieu Ferrari, Saket Srivastava, Ram Jha, Frederick Arce Vargas, Kevin Duffy, William Day, Paul Virgo, Lucy Wheeler, Jeremy Hancock, Farzin Farzaneh, Sabine Domning, Yiyun Zhang, Nushmia Z. Khokhar, Vijay G. R. Peddareddigari, Robert Wynn, Martin Pule, Persis J. Amrolia

**Affiliations:** 1 Autolus PLC, London, UK; 2 Department of Haematology, University College London Hospitals NHS Trust, London, UK; 3 Departments of Bone Marrow Transplant and Haematology, Great Ormond Street Hospital for Children, London, UK; 4 Department of Blood and Marrow Transplant, Royal Manchester Children’s Hospital, Manchester, UK; 5 Department of Immunology and Immunogenetics, North Bristol NHS Trust, Bristol, UK; 6 Bristol Genetics Laboratory, North Bristol NHS Trust, Bristol, UK; 7 Rayne Institute, Kings College London, London, UK; 8 Cancer Institute, University College London, London, UK

**Keywords:** Acute lymphocytic leukaemia, Paediatric cancer, Cancer immunotherapy, Phase I trials, Immunotherapy

## Abstract

Chimeric antigen receptor (CAR) T cells targeting CD19 or CD22 have shown remarkable activity in B cell acute lymphoblastic leukemia (B-ALL). The major cause of treatment failure is antigen downregulation or loss. Dual antigen targeting could potentially prevent this, but the clinical safety and efficacy of CAR T cells targeting both CD19 and CD22 remain unclear. We conducted a phase 1 trial in pediatric and young adult patients with relapsed or refractory B-ALL (*n* = 15) to test AUTO3, autologous transduced T cells expressing both anti-CD19 and anti-CD22 CARs (AMELIA trial, EUDRA CT 2016-004680-39). The primary endpoints were the incidence of grade 3–5 toxicity in the dose-limiting toxicity period and the frequency of dose-limiting toxicities. Secondary endpoints included the rate of morphological remission (complete response or complete response with incomplete bone marrow recovery) with minimal residual disease-negative response, as well as the frequency and severity of adverse events, expansion and persistence of AUTO3, duration of B cell aplasia, and overall and event-free survival. The study endpoints were met. AUTO3 showed a favorable safety profile, with no dose-limiting toxicities or cases of AUTO3-related severe cytokine release syndrome or neurotoxicity reported. At 1 month after treatment the remission rate (that is, complete response or complete response with incomplete bone marrow recovery) was 86% (13 of 15 patients). The 1 year overall and event-free survival rates were 60% and 32%, respectively. Relapses were probably due to limited long-term AUTO3 persistence. Strategies to improve CAR T cell persistence are needed to fully realize the potential of dual targeting CAR T cell therapy in B-ALL.

## Abstract (web summary)

Bicistronic CAR T cells targeting CD19 and CD22 exhibit clinical activity and low toxicity in pediatric and young adult patients with B cell acute lymphoblastic leukemia, with relapses associated with limited CAR T cell persistence.

## Main

CD19 and CD22 chimeric antigen receptor (CAR) T cell therapies have shown promising efficacy in relapsed or refractory B-lineage acute lymphoblastic leukemia (B-ALL). However, CD19-negative relapse is the predominant cause of treatment failure in patients treated with anti-CD19 CAR T cells as a standalone therapy, occurring in 25–42% of responding patients^1,2^. Similarly, reduced CD22 antigen density at relapse was observed in a phase 1 study of anti-CD22 CAR T cell therapy, suggesting that escape by CD22 downregulation is also possible^3,4^. We reasoned that dual antigen targeting may prevent relapse given that a single leukemic stem cell is unlikely to downregulate both CD19 and CD22 simultaneously. Dual CAR targeting can be achieved in different ways: co-administration of two separate CAR T cell products (CD19 CAR T cells and CD22 CAR T cells), co-transduction of T cells with two vectors encoding the two separate CARs, transduction of T cells with a bicistronic vector encoding both CARs, or use of a tandem CAR. The optimal strategy is yet to be defined: each has its strengths and weaknesses and all are currently under investigation^5–7^. Although co-administration or co-transduction allows for the combination of two single targeting vectors with minimal optimization, it results in a heterogeneous mixed product that has a more complex and costly manufacturing procedure. In contrast, bicistronic vectors encoding both CARs, and tandem CAR approaches are cheaper to manufacture and generate a more uniformed homogeneous product that ensures that every transduced cell has the ability to engage both targets.

We developed AUTO3, a CAR T cell treatment with dual specificity generated through transduction of autologous T cells with a bicistronic γ-retroviral vector encoding humanized anti-CD19 and CD22 CARs. Both CARs are in second-generation format and incorporate tumor necrosis factor receptor (TNFR) co-stimulatory domains. The CD22 CAR functionality was further enhanced through the incorporation of a pentameric coiled-coil spacer from cartilage oligomeric matrix protein (COMP). Pre-clinical experiments confirmed the ability of AUTO3 to target both double- and single-positive targets with superior in vitro and in vivo cytotoxicity over a CD19 CAR with the FMC63 binder used in tisagenlecleucel.

Based on the results of the pre-clinical data, we designed a phase 1 clinical study with AUTO3 in pediatric and young adult patients with relapsed or refractory B-lineage ALL (AMELIA trial; NCT03289455, EUDRA CT 2016-004680-39). Here, we report the pre-clinical experiments with AUTO3 together with the safety and efficacy results of this phase 1 clinical trial.

## Results

### Recognition of both CD19 and CD22 by AUTO3 CAR T cells

**Fig. 1:**
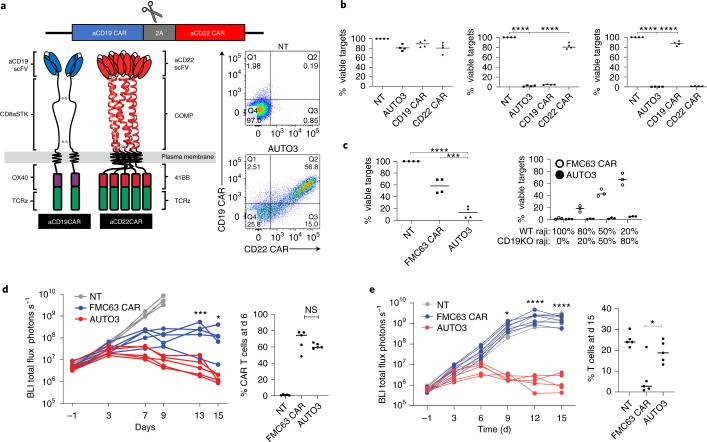
Pre-clinical validation of AUTO3. **a**, AUTO3 is a dual CD19 and CD22 CAR T cell encoded on a single bicistronic γ-retroviral vector under the same promoter. The two CARs are separated by a self-cleaving 2A peptide that allows for equal expression, as shown in the flow cytometry plot. NT, non-transduced. **b**, Cytotoxicity of T cells co-cultured for 72 h with NT (left), CD19^+^ (middle) or CD22^+^ (right) SupT1 targets at a 1:1 effector : target (E:T) ratio. Data were normalized to NT T cells (*n* = 4 biologically independent samples). **c**, Comparison of cytotoxicity between AUTO3 and FMC63 CAR against Raji tumor cell lines. (left) Cytotoxicity of AUTO3 and FMC63 CAR T cells co-cultured with Raji for 24 h at a 1:1 E:T ratio (*n* = 4 biologically independent samples). (right) Cytotoxicity against mixed targets of WT Raji and CD19KO Raji cells at a 1:1 E:T for 72 h (*n* = 3 biologically independent samples). **b**,**c**, Horizontal lines represent the median of data points from healthy individual donors. ****P < 0.0001, ***P = 0.0001, one-way analysis of variance (ANOVA) with Dunnett’s post hoc test for multiple comparisons. **d**, In vivo efficacy of AUTO3 in a Nalm6 NSG mouse model after treatment with 5 × 10^6^ CAR T cells. BLI, bioluminescent imaging. (left) Total flux radiance of tumor engraftment kinetics 15 d after treatment with CAR T cells (*n* = 5 FMC63 CAR and AUTO3, *n* = 3 NT, biologically independent animals). ***P = 0.0006, *P = 0.0437, two-way ANOVA with Bonferroni correction between FMC63 and AUTO3 over all six time points. (right) Percentage of CAR T cells in the bone marrow 6 d after CAR treatment (*n* = 5 biologically independent animals). Horizontal lines represent the median. *P* = 0.260 (NS, not significant), unpaired *t*-test. **e**, In vivo efficacy of AUTO3 in a CD19KO Nalm6 NSG model after treatment with 5 × 10^6^ CAR T cells. (left) Tumor engraftment kinetics over 15 d (*n* = 5 biologically independent animals). ****P < 0.0001, *P = 0.0304, two-way ANOVA with Bonferroni correction between FMC63 and AUTO3 over all six time points. (right) CAR T cells in the bone marrow at d 15 (*n* = 5 biologically independent animals). Horizontal lines represent the median. *P = 0.0216, unpaired *t*-test.

**Extended Data Fig. 1:**
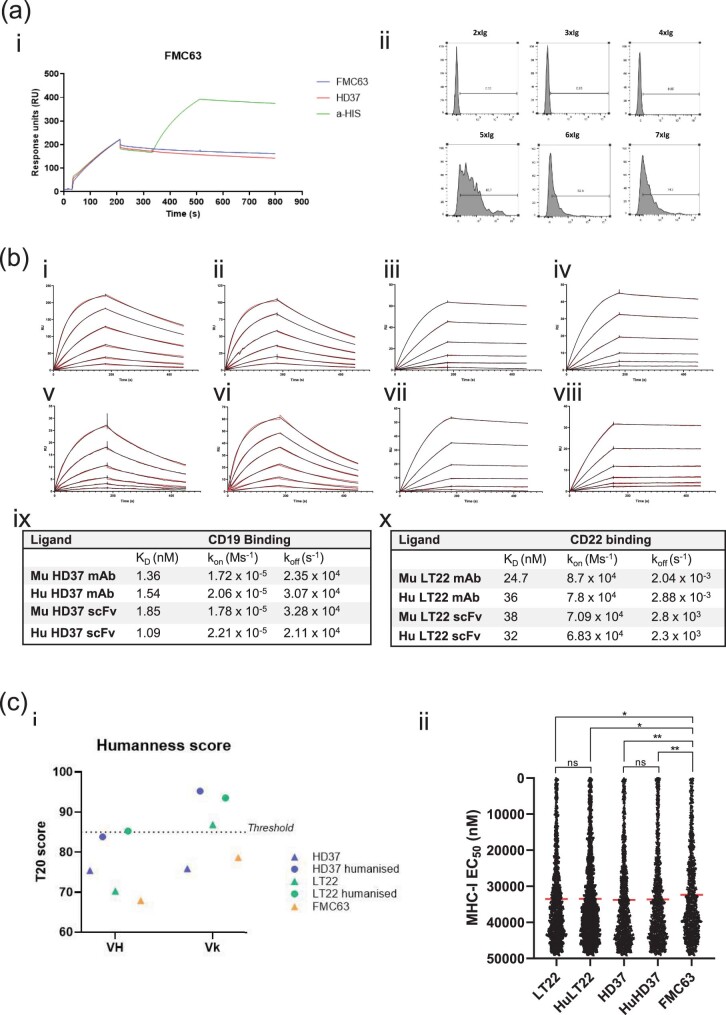
Biophysical evaluation of the CD19 and CD22 binders. **a)** Competition for binding to CD19 between scFv proteins. FMC63 was captured as the ligand and injections of recombinant CD19 followed by FMC63 scFv, HD37 scFv or anti-His tag Ab were used as analyte (i). HD37 did not show interaction with CD19 bound to FMC63, indicating a shared or overlapping epitope. As expected, FMC63 was also unable to bind CD19, while the anti-His recognised a distinct epitope at the C-term of CD19. (ii) Determination of CD22 antibody binding epitope. LT22 was tested against various truncated variants of CD22. Binding was observed upon expression of Ig domain 5 indicating that the antibody targets this region. **b)** Humanisation of CD19 and CD22 binding domains does not affect binding kinetics in Surface Plasmon Resonance assay. Binding of Murine LT22 mAb (i), Humanised LT22 mAb (ii), Murine LT22 scFv (iii) and HuLT22 scFv (iv) to CD22. Binding of Murine HD37 mAb (v), Humanised HD37 mAb (vi), Murine HD37 scFv (vii) and HuHD37 scFv (viii) to CD19. i-viii) Black = reference subtracted sensograms; Red = sensogram fit with 1:1 Langmuir binding model. (ix) binding kinetic values for murine and humanized anti-CD19 mAb and scFv for CD19. (x) binding kinetic values for murine and humanized anti-CD22 mAb and scFv. **c)** Antibody humanness T20 score for the VH and Vk sequence of HD37, humanised HD37, LT22, humanised LT22 and FMC63 (i). A score >85 is associated with a human sequence. Humanised HD37 and LT22 binders show improved scores, with values nearing or surpassing the humanness threshold. (ii) Prediction of Human MHC-I affinity (EC50) on HLA allele A*02:01 for scFv-derived peptides (8-14 aa). Red line = mean value of individual datasets. No significant average MHC-I affinity difference was determined for humanised HD37 (P = 0.3799) and LT22 (P = 0.9268) binders compared to their respective parental antibody (unpaired t-test). Significantly lower average MHC-I affinity was determined for both HD37 and LT22 (humanised and parental), compared to FMC63, suggesting reduced immunogenicity. One-way ANOVA (Dunnett post-test) *P = 0.0250 for FMC63 vs. LT22, *P = 0.0322 for FMC63 vs. HuLT22, **P = 0.0040 for FMC63 vs. HD37, **P = 0.0088 for FMC63 vs. HuHD37. Unpaired t test P = 0.9268 for LT22 vs. HuLT22 and P = 0.8150 for HD37 vs. HuHD37.

AUTO3 is a dual CAR T cell treatment generated through the transduction of autologous T cells with an RD114 pseudotyped, bicistronic γ-retroviral vector encoding a CD19 and CD22 CAR separated by a self-cleaving 2A peptide, transcribed from a single promoter (Fig. 1a). The CD19-binding moiety was derived from the HD37 antibody and targets a dominant epitope on the protein that is shared with other CD19-binding antibodies including FMC63; the CD22-binding moiety was derived from the LT22 antibody and targets the Ig-like C2-type 5 domain (Extended Data Fig. 1a). In an effort to reduce the immunogenicity of the AUTO3 product, both CD19 and CD22 binders were humanized in a process that maintained similar biophysical properties as the parental murine binders (Extended Data Fig. 1b,c).

**Extended Data Fig. 2:**
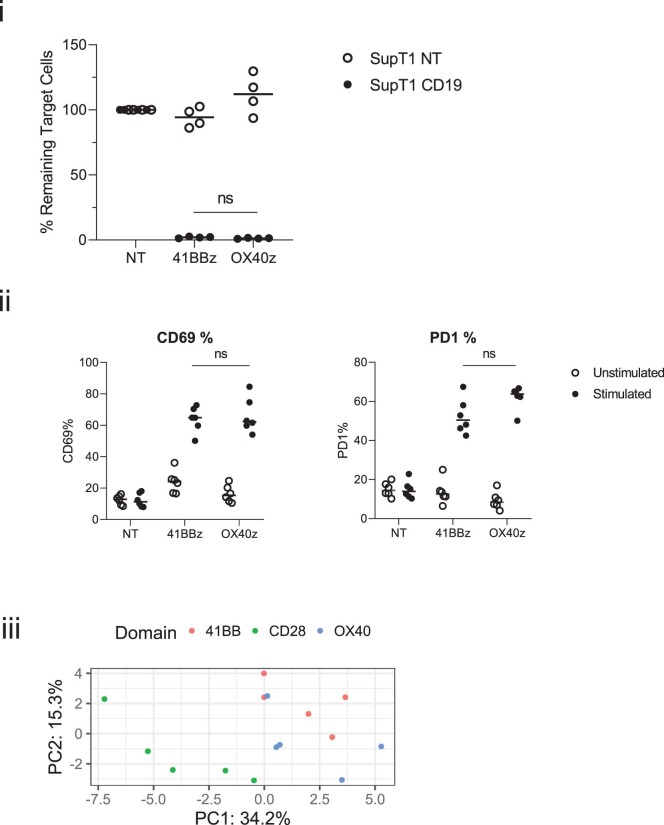
Comparison of OX40 and 41BB function. PBMCs transduced to express the anti-CD19 CAR HD37 with either a 41BBz or OX40z endodomain. i) CAR T cells were co-cultured at a 1:1 ratio with either non-transduced SupT1 targets cells (NT) or SupT1 cells transduced to express CD19. After 72 h the remaining targets cells were assessed by flow cytometry. n = 4 biologically independent samples, statistical analysis performed by one-way ANOVA with Dunnett’s post-test comparing the 41BBz CAR to all other conditions. ns = non-significant, P = 0.1078. ii) 1×10^6^ transduced CAR T cells were stimulated with 50ug/ml HD37 idiotype for 24 h before assessing the expression activation markers CD69 and PD1 by flow cytometry. Lines represent the median value, n = 6 biologically independent samples. Statistical analysis performed by one-way ANOVA with Dunnett’s post-test comparing the 41BBz CAR to all other conditions. ns = non-significant, CD69% - P = 0.8009 and PD1% - P = 0.0730. iii) 1×10^6^ transduced CAR T cells were stimulated with 50ug/ml HD37 idiotype for 24 h. RNA was extracted from stimulated CAR T cells and gene profile assessed using the nCounter CAR T cell characterisation panel (Nanostring). The panel of immune persistence genes were analysed via Principal Component Analysis (PCA) using R/stats version 4.0.5 (n = 5 healthy donors).

The TNFR co-stimulatory domains were used based on their ability to maintain engraftment of the CAR T cells compared with CD28 co-stimulation domains^8,9^. The CD19 CAR incorporates an OX40 co-stimulatory domain and the CD22 CAR incorporates a 41BB domain (Fig. 1a). OX40 has an analogous immune function to 41BB (ref. ^10^) and utilizes similar downstream TNFR-associated factor (TRAF) signaling molecules^11,12^. A comparison of CD19 CARs encoding either OX40 or 41BB co-stimulation domains showed equivalent function and upregulation of activation markers, with a more similar activated gene expression profile between OX40 and 41BB than compared with a CD28 encoding CAR (Extended Data Fig. 2).

**Extended Data Fig. 3:**
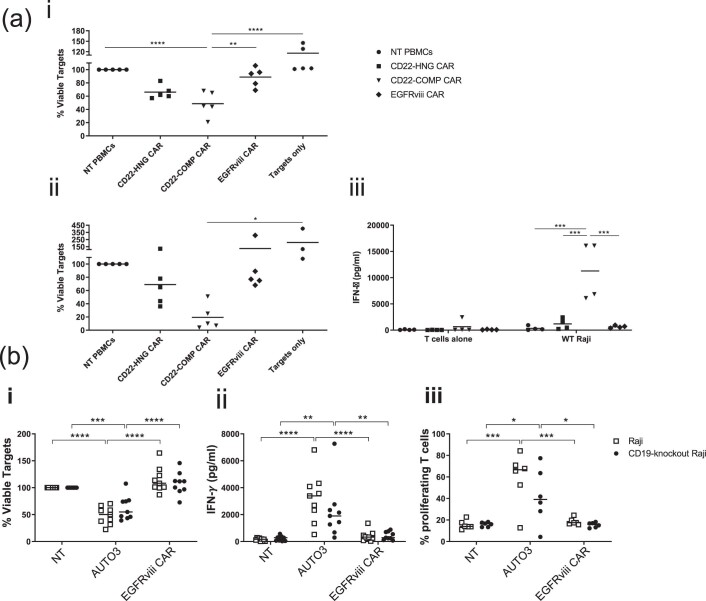
In vitro activity of CD19/CD22 CARs. **a)** Comparison of CD22CAR cytotoxicity and cytokine secretion with two different spacers. The spacers domains from a pentameric coiled-coil spacer from cartilage oligomeric matrix protein (CD22-COMP CAR) or the hinge domain from human IgG1 (CD22-HNG CAR) were used to transduced T cells. A Cytotoxicity was determined after 72 h co-cultures with Raji (i) and SupT1 CD22+ (ii) tumour cell lines (1:1 E:T). EGFRvIII CAR was used as an irrelevant CAR and data were normalised to non-transduced (NT) T cells. iii) Cytokine release of CAR T cells co-cultured with Raji (1:1 E:T at 72 h). Lines represent the mean of individual healthy donors. Statistical analysis performed by one-way ANOVA with Dunnett’s post-test comparing the CD22 COMP-CAR to all other conditions (n = 3-5 biologically independent samples). (i) ****P < 0.0001, **P = 0.0012, (ii) *P = 0.012, (iii) ***P = 0.0004 for NT PBMCs vs CD22 COMP-CAR, ***P = 0.0008 for CD22 HNG-CAR vs CD22 COMP-CAR, ***P = 0.0005 for EGFR viii CAR vs CD22 COMP-CAR. **b)** Activity of CD19/CD22 CAR against CD19 ^−^ negative tumor cells. Cytotoxicity (i) and IFN-γ release (ii) was assessed after AUTO3 CAR T cells were co-cultured for 24 hrs with Raji (empty squares) or CD19 ^−^ knockout Raji cells (solid dots) at a 1:1 E:T. EGFRvIII CAR was used as an irrelevant CAR and cytotoxicity data were normalised to non-transduced (NT) T cells (n = 9 biologically independent samples). iii) Proliferation: Transduced T cells prepared as above were labelled with CTV (Cell Trace Violet) prior to 1:1 co-culture with Raji or CD19 ^−^ knockout Raji cells. After 4 days the fluorescence of CTV on CD3+ cells was measured by flow cytometry. Lines represent the mean value of individual healthy donors (n = 6 biologically independent samples). Statistical analysis performed by one-way ANOVA with Dunnett’s post test comparing AUTO3 CAR against NT and EGFRviii CAR within each target group (Raji or CD19 ^−^ KO Raji); (i), (ii) and (iii) ****P < 0.0001; (i) ***P = 0.0003; (ii) **P = 0.003 for AUTO3 vs NT and **P = 0.007 for AUTO3 vs EGFRvIII CAR; (iii) ***P = 0.0005 for AUTO3 vs EGFRvIII CAR and ***P = 0.0002 for AUTO3 vs NT, *P = 0.017 for AUTO3 vs NT and *P = 0.015 for AUTO3 vs EGFRvIII CAR.

To enhance CD22 targeting, the CD22 CAR used the pentameric coiled-coil domain from COMP as a CAR spacer, which resulted in improved sensitivity to CD22 (Extended Data Fig. 3a). Functional testing of AUTO3 showed specific killing of engineered cell lines expressing CD19 alone or CD22 alone (Fig. 1b). In an in vitro experiment mimicking the emergence of CD19 antigen escape of a Burkitt’s lymphoma cell line, AUTO3 was capable of effectively killing, inducing interferon (IFN)-γ secretion and proliferating in response to CD19^−^/CD22^+^ Raji targets (Extended Data Fig. 3b).

### Superiority of AUTO3 over an FMC63-based CAR

**Extended Data Fig. 4:**
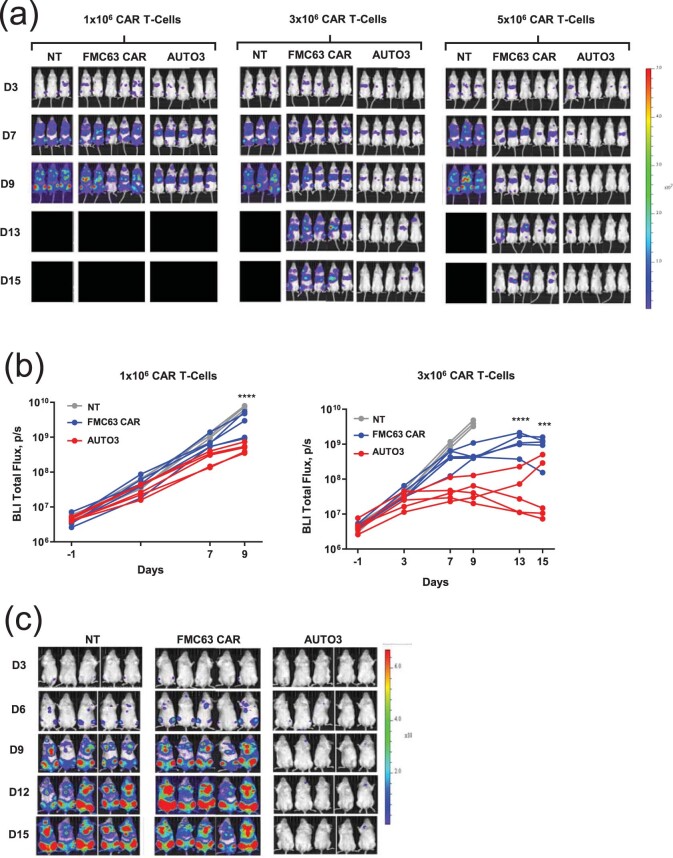
In vivo efficacy of AUTO3 in Nalm6 NSG models. In vivo efficacy of AUTO3 in Nalm6 NSG models. (a-c) CD19+ CD22+ Nalm6 cells transduced to express luciferase were IV administered into NSG mice. After 4 days, 1×10^6^, 3×10^6^ or 5×10^6^ transduced AUTO3 CAR T cells or matched number of non-transduced (NT) T cells were administered IV. a) Bioluminescent imaging (BLI) of the Nalm6 tumor burden measured post CAR T-cell injection. b) The mean total flux radiance quantified pre (day -1) and post administration of 1×10^6^ and 3×10^6^ CAR T-cells (n = 5 for FMC63 and AUTO3, n = 3 for NT). c) BLI of a CD19 knockout (CD19KO) Nalm6 NSG model. NSG mice were IV injected with luciferase positive CD19KO Nalm6 cells 8 days prior to the IV administration of 5×10^6^ transduced CAR T-cells or matched number of non-transduced (NT) T cells (n = 5 biologically independent animals). Statistical analysis performed by two-way ANOVA with Bonferroni correction between FCM63 and AUTO3 over all time points. 1×10^6^ dose **** P = 0.00002. 3×10^6^ dose day 13 ****P < 0.00001, day 16 *** P = 0.00057.

When AUTO3 was compared with a CD19 FMC63-based 41BB-Z CAR used in tisagenlecleucel, AUTO3 showed enhanced cytotoxicity against CD19^+^/CD22^+^ Raji cells in the in vitro co-cultures (*P* = 0.0001, Fig. 1c). When cytotoxicity assays were performed with increasing proportions of CD19^−^/CD22^+^ targets, CAR T cells expressing an FMC63-based CD19 CAR showed progressive reduction in cytotoxicity whereas AUTO3 generated from the same donor highly effectively killed both CD19^+^ and CD19^−^ Raji targets (Fig. 1c). In a NOD/SCID/ɣ (NSG) mouse CD19^+^/CD22^+^ tumor model, AUTO3 demonstrated improved efficacy over the FMC63 CAR with equivalent persistence in the bone marrow (Fig. 1d). Furthermore, this superior efficacy was maintained at lower, suboptimal doses of CAR T cells (Extended Data Fig. 4). In a model recapitulating the loss of CD19 (CD19^−^/CD22^+^ tumor), AUTO3 CAR T cells were able to stop tumor growth and reduce tumor burden and had a higher CAR T cell persistence in the bone marrow compared with the FMC63 CAR, which showed no improvement over non-transduced T cells (Fig. 1e and Extended Data Fig. 4).

### Phase 1 trial with AUTO3

In the dose-finding portion of the AMELIA trial, pediatric or young adult patients with relapsed or refractory B-ALL (aged 1–24 years) were enrolled between 28 July 2017 and 10 June 2019 in three UK centers. Although originally planned, the adult cohort (aged ≥ 25 years) was not initiated. The initial study design included a phase 2 expansion cohort in the pediatric and young adult patient population to further evaluate AUTO3 efficacy. A study amendment in June 2019 included a phase 1 cohort for adult patients (at the highest dose level declared safe in the pediatric and young adult patient population). The study closure occurred before the end of the dose escalation phase. Study design and eligibility criteria are outlined in the Methods section.

The primary endpoints of the study were the incidence of grade 3–5 toxicity in the dose-limiting toxicity (DLT) period (30 d after AUTO3 infusion) and the frequency of DLTs. Secondary endpoints included the proportion of patients achieving morphological remission (complete response or complete response with incomplete bone marrow recovery) and having a minimal residual disease (MRD)-negative response after AUTO3 infusion, as well as the frequency and severity of adverse events, the expansion and persistence of AUTO3, the duration of B cell aplasia, and overall and event-free survival.

**Fig. 2:**
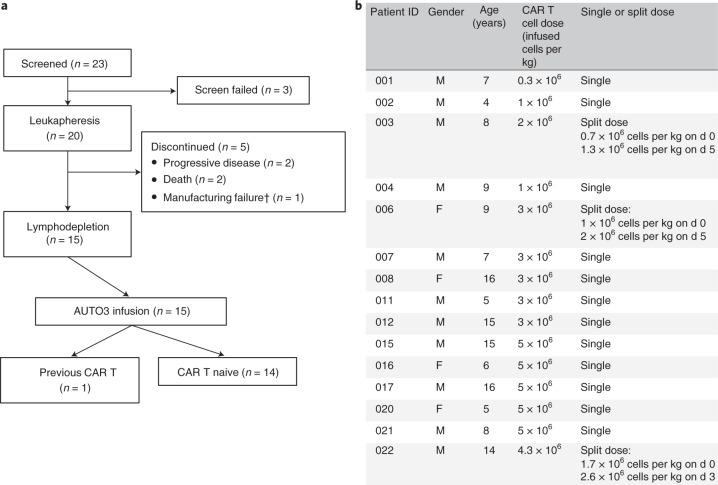
CONSORT diagram and patient CAR T cell dosing. **a**,**b**, CONSORT flow diagram (**a**) and the total AUTO3 dose and schedule (single versus split dose) for each patient treated with AUTO3 (**b**). Of the 20 patients who underwent leukapheresis, 15 received AUTO3 because two patients died, two discontinued due to progressive disease before the start of any study treatment and one AUTO3 final product was not released by quality control (^†^classified as manufacturing failure). CAR T cells were infused as a single dose on d 0 for 12 of the 15 patients. Three patients received a dose that was split. For one patient the dose was split between d 0 and d 3. For two patients the dose was split between d 0 and d 5. Target doses of 1 × 10^6^, 3 × 10^6^ and 5 × 10^6^ cells per kg were explored. There were three outliers: patient 001 received a total of 0.3 × 10^6^ cells per kg because after the first dose a serious adverse event (encephalopathy) occurred and prevented him from receiving the second dose of 0.7 × 10^6^ cells per kg; patient 003 was enrolled in the 1 × 10^6^ cells per kg cohort and 2 × 10^6^ cells per kg were manufactured, but the 3 × 10^6^ cells per kg cohort was opened at the time of dosing and he therefore received the full dose available (2 × 10^6^ cells per kg); and patient 022 had a poor leukapheresis product (>70% blasts), and Autolus was able to manufacture 4.3 × 10^6^ cells per kg. F, female; M, male.

**Fig. 3:**
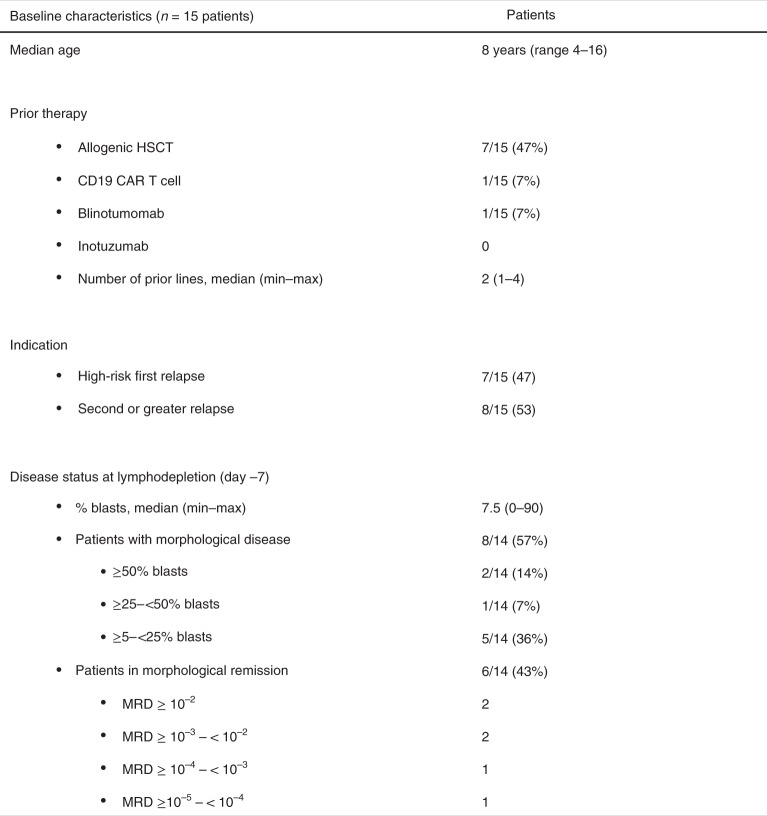
Baseline characteristics of the patients at enrollment. The leukemia disease burden was assessed on the day before the start of the lymphodepletion. HSCT, hematopoietic stem cell transplantation.

**Extended Data Fig. 5:**
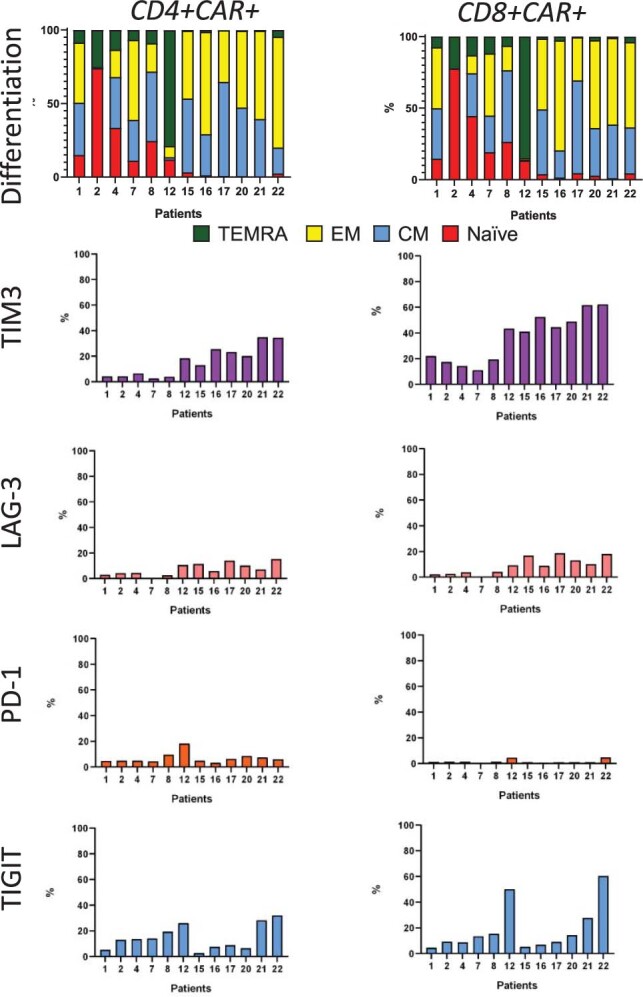
AUTO3 product phenotype characterization data. Drug product memory phenotype composition as percentage of CD4+ or and CD8+ CAR T cells: naïve T cells (CD45RA+, CCR7+), central memory (CM) T cells (CD45RA-, CCR7+), effector memory (EM) T cells (CD45RA-, CCR7-), terminally differentiated T effector memory cells re-expressing CD45RA, TEMRA (CD45RA+, CCR7-). Drug product exhaustion phenotype composition based on percentage of CD4+ or CD8+ CAR T cells expressing TIM3, LAG3, PD-1 or TIGIT. Stacked bars show percentage of cells expressing single markers per individual patients.

A total of 23 patients were screened, a product was generated in 19 of 20 patients (95%) who underwent leukapheresis, and a total of 15 patients received infusion of AUTO3 (Fig. 2). Patients were followed up to a data cut-off of 1 June 2020, with a median follow-up of 14 months (range, 2–28 months). The median age was 8 years (range, 4–16 years) and the median number of prior lines of therapy was 2 (range, 1–4). One patient had previously received CD19 CAR T cell and blinatumomab therapy, while the remaining 14 patients were CAR T cell naive. Seven of 15 patients had relapsed following allogeneic stem cell transplant, and seven and eight patients were considered as having high-risk first relapse and second or greater relapse, respectively (Fig. 3). AUTO3 CAR T cells were manufactured using a semi-automated process on the CliniMACS Prodigy. The median transduction efficiency was 17.7% (range, 8.6–39.3%) and the median vector copy number in the product was 0.55 per cell (range, 0.26–1.46 per cell). AUTO3 showed a predominantly effector memory phenotype, with a lesser contribution of central memory cells and a low expression of exhaustion markers in most patients (Extended Data Fig. 5).

Prior to lymphodepletion, of 14 patients with evaluable morphological bone marrow assessment, eight were in morphological relapse (5–90% blasts) and six had MRD-level disease. None of the 15 patients had concomitant extramedullary disease. As per the protocol, all 15 patients received pre-conditioning with 30 mg m^−2^ d^−1^ fludarabine for 4 d and 500 mg m^−2^ d^−1^ cyclophosphamide for 2 d prior to AUTO3 infusion. The study was designed as a dose escalation: four patients received 0.3–2 × 10^6^ CAR^+^ T cells per kg body weight, five received 3 × 10^6^ cells per kg and six received 4.3–5 × 10^6^ cells per kg (Fig. 2b). The high-dose cohort (10 × 10^6^ cells per kg body weight) was not initiated. To mitigate the risk of severe toxicity in patients with high disease burden, and depending on the leukemia burden in the bone marrow on the day before initiation of lymphodepletion (d −7), CAR T cells were infused as a single dose (<25% blasts) or as a split dose (≥25% blasts).

### AUTO3 toleration with mild toxicity

**Fig. 4:**
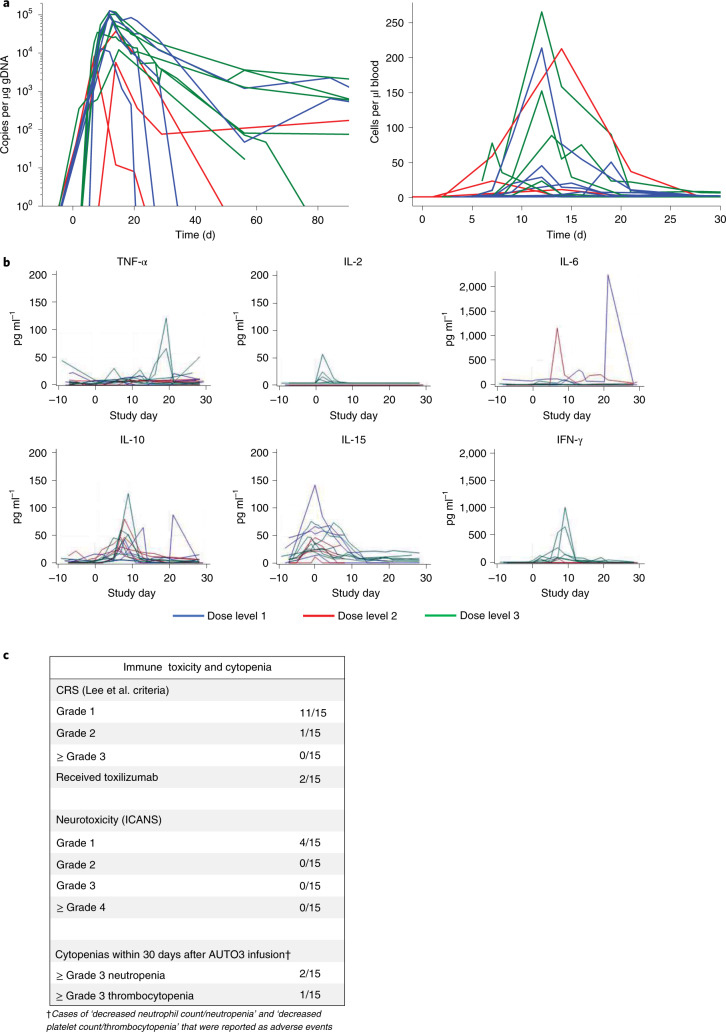
Pharmacodynamics, serum cytokines and adverse events. **a**, CAR T cell marking for all patients with evaluable peripheral blood samples. Copies per μg genomic DNA as detected using real-time PCR (left) in the first 90 d after infusion, and marking by flow cytometry (right) using an anti-CD19 CAR anti-idiotype as CAR T cells per μl blood in the first 30 d after infusion. **b**, Serum cytokines for all patients in the first 30 d after CAR T cell infusion. **c**, Immune toxicity and reported adverse events of cytopenias within 30 d after AUTO3 infusion.

**Extended Data Fig. 6:**
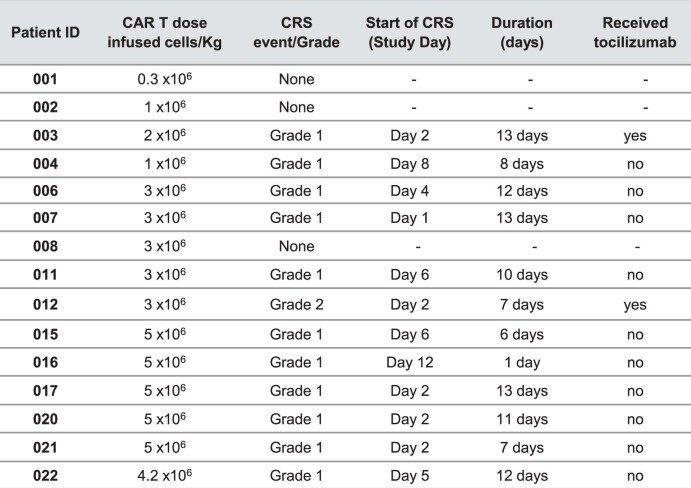
CRS events per patient. Duration defined as day from start of 1^st^ selected AE to end of last AE related to CRS by the investigator. Selected AEs included: febrile neutropenia, hypotension, pyrexia, tachycardia. Neutropenia alone was not considered a CRS related AE and was excluded from this analysis. No patients were admitted to intensive care unit (ICU) due to CRS.

**Extended Data Fig. 7:**
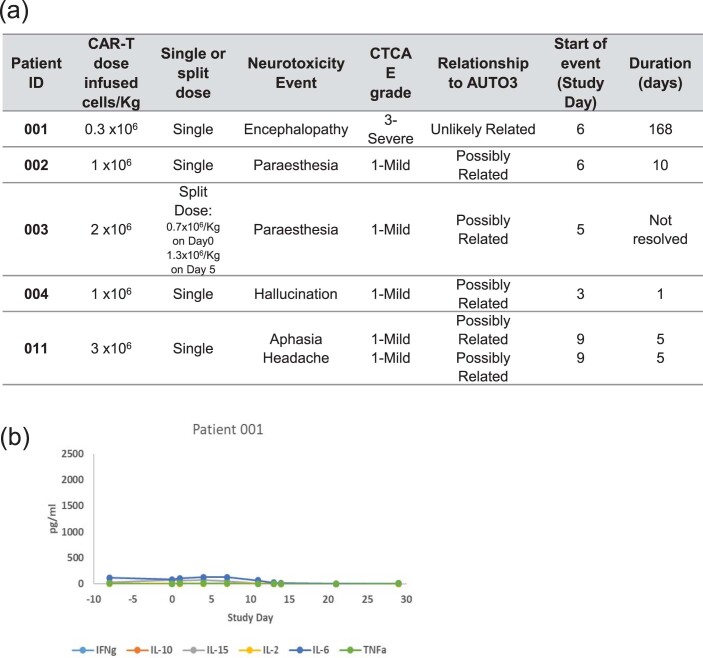
Neurotoxicity. **a)** Summary of neurotoxicity events in the 5 patients who had any reported neurotoxicity AE. Note: Patient 3 had a not resolved event because the event was still ongoing when patient died due to disease progression 2.3 months after AUT03 infusion**. b)** Serum cytokine levels in patient 001 who experienced encephalopathy 5 days after a 0.3×10^6^cell/kg dose of AUTO3 infusion, the encephalopathy was considered likely related to the prior infusion of intrathecal methotrexate and unlikely related to AUTO3.

During the dose escalation up to 5 × 10^6^ cells per kg, grade 3–5 toxicity was seen in 9 of 15 patients (60%) and no DLTs were reported. The frequency of DLTs was a primary endpoint of the trial. A maximum tolerated dose was not reached. Twelve of 15 patients (80%) developed cytokine release syndrome (CRS) at a median of 3 d (range, 1–12 d) after AUTO3 infusion. CRS was mild in all cases (grade 1, *n* *=* 11 patients; grade 2, *n* *=* 1 patient as assessed with the Lee et al.^13^ criteria). No grade 3–4 CRS was observed (Fig. 4c). Only two patients were treated with tocilizumab and no patients required admission to intensive care due to CRS (Extended Data Fig. 6). In concordance with the absence of severe CRS, elevation of serum levels of interleukin (IL)-6, IL-10, IFN-γ and tumor necrosis factor (TNF)-α was modest (Fig. 4b) compared with reported data with tisagenlecleucel^14^. Four patients had AUTO3-related neurotoxicity, grade 1 in all, with symptoms including headache, paresthesia, aphasia and hallucination (Fig. 4c and Extended Data Fig. 7a). One patient developed grade 3 encephalopathy 6 d after a single dose of 0.3 × 10^6^ cells per kg AUTO3, which was probably related to the prior treatment with intrathecal methotrexate. The presence of initial neurological symptoms prior to the CAR T cells infusion, the complete absence of CRS symptoms, the very low levels of proinflammatory cytokines and the additional appearances of atypical therapy-related leukoencephalopathy on magnetic resonance imaging suggest that this was unlikely to be CAR-associated neurotoxicity (Extended Data Fig. 7b).

**Extended Data Fig. 8:**
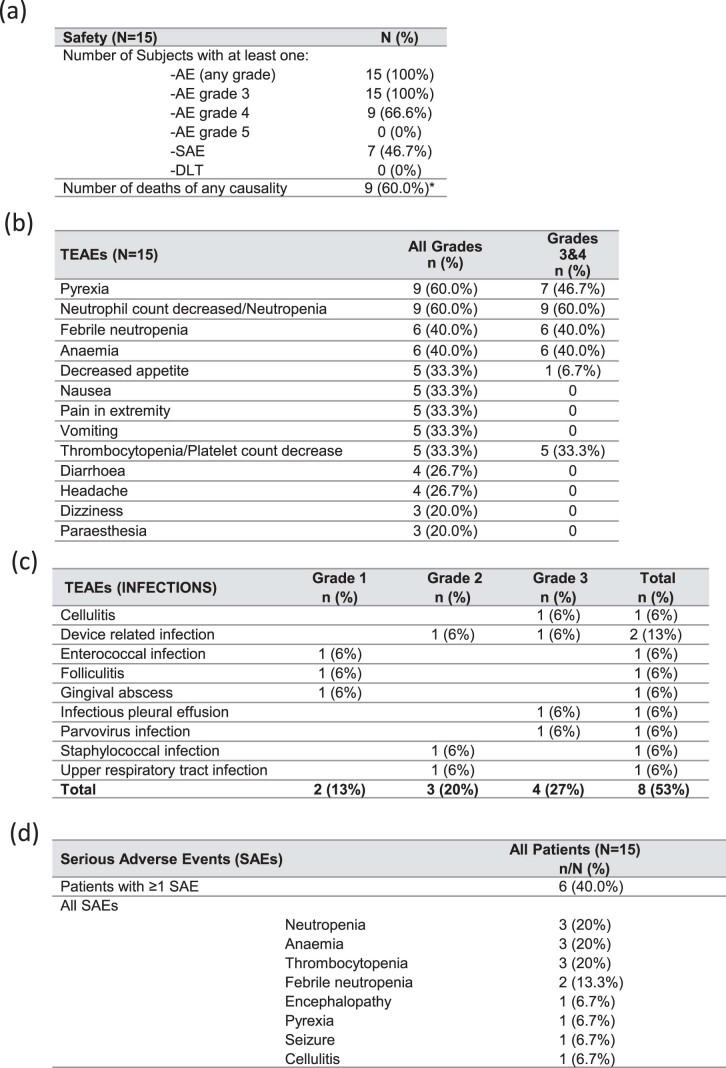
General Toxicity. **a)** Summary table of adverse events by grade (occurring anytime from AUTO3 infusion until the data cut-off), SAEs, DLTs and deaths. *All deaths were due to disease progression. **b)** Treatment-Emergent Adverse Events (TEAEs) by preferred term with incidence of ≥ 20%. Treatment-emergent was defined as any AE that occurred during or after administration of AUTO3 and anytime up to the data cut-off **c)** Treatment emergent infections by grade and infection type. No Grade 4 or 5 infections were reported. **d)** Serious adverse events, all patients and SAEs occurring in ≥5% patients (occurring anytime from AUTO3 infusion until the data cut-off).

Nine of the 15 patients (60%) developed grade 3–4 toxicity by d 30, including fever (*n* = 7 patients), febrile neutropenia (*n* = 4 patients) neutropenia (*n* = 2 patients), anemia (*n* = 2 patients), thrombocytopenia (*n* = 1 patient) and infections (*n* = 2 patients). No deaths due to adverse events were observed. Overall, the most common grade 3–4 treatment-emergent adverse events (at any time after AUTO3 infusion) were neutropenia (9 of 15 patients), fever (7 of 15 patients), febrile neutropenia (6 of 15 patients), anemia (6 of 15 patients) and thrombocytopenia (5 of 15 patients). The incidence of grade 3 infections was 27% (4 of 15 patients) and no grade 4 infections were reported. Safety summary, treatment adverse events and serious adverse events are given in Extended Data Fig. 8.

### Expansion of AUTO3 with limited long-term persistence

**Extended Data Fig. 9:**
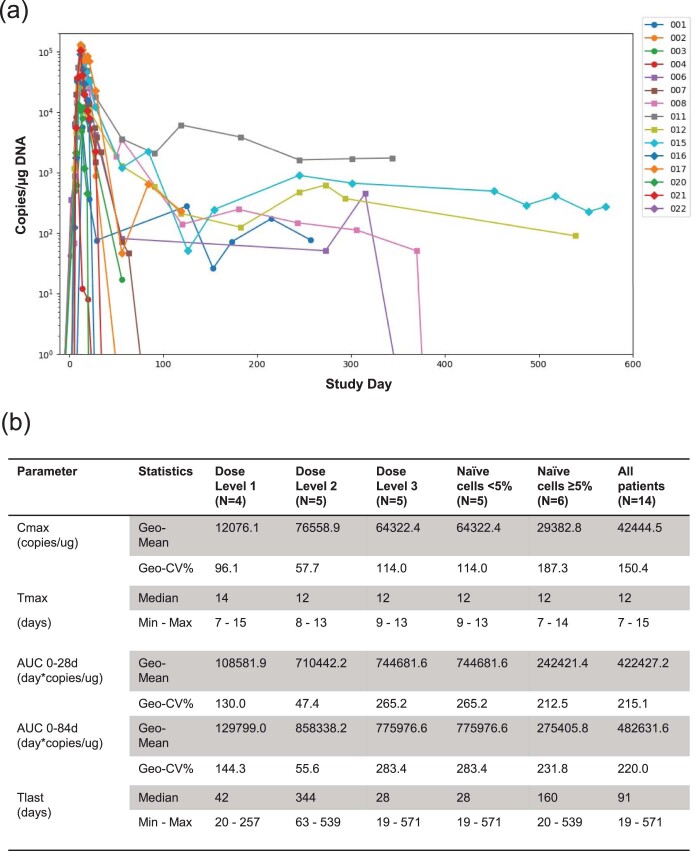
CAR T engraftment kinetics. **a)** Individual concentration-time profiles for AUTO3 transgene levels by qPCR in peripheral blood post AUTO3 infusion. **b)** Summary of CAR T cell kinetic parameters as measured in peripheral blood by qPCR. Cmax - maximum concentration; Tmax - Time to Cmax (Days), Tlast -Time to last measurable in blood (Days), AUC - area under the curve; AUC 0-28d - AUC from time zero to day 28; AUC 0-84d - AUC from time zero to day 84; Time to Cmax is the time to reach peak CAR T cell concentration. Dose Level 1, 0.3-2 ×10^6^ cells/kg, Dose Level 2, 3×10^6^ cells/kg, Dose level 3, 4.3-5 ×10^6^ cells/kg. Of Note: 1 non-responding patient did not have any CAR-T expansion and therefore was non-evaluable for kinetic parameter derivation.

Substantial peripheral CAR T cell expansion (maximum concentration (C_max_) > 30,000 copies per µg DNA) was seen in 10 of 15 patients (66%) (Fig. 4a), with a median time to maximal expansion of 12 d (range, 7–15 d) and a mean area under the curve (AUC) from d 0 to d 28 (AUC_d0–28_) of 422,427 copies per µg DNA. Of the 13 patients who achieved complete response or complete response with incomplete bone marrow recovery, the kinetics of initial AUTO3 expansion were similar to that previously described for tisagenlecleucel^2^ (mean C_max_, 46,717 versus 36,100 copies per μg DNA; AUC_d0–28_, 453,477 versus 315,000 copies per μg DNA in the patients who responded to AUTO3 and tisagenlecleucel, respectively) (Extended Data Fig. 9b). CAR T cell expansion in the two non-responding patients was poor.

**Extended Data Fig. 10:**
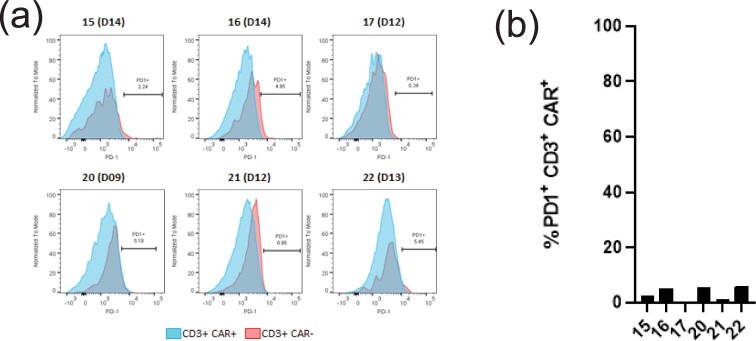
PD-1 expression at peak CAR expansion. **a)** Expression of PD1 on CD3+ CAR+ cells from peripheral blood at the peak of expansion analyzed by flow cytometry in patients with the highest dose cohort. Treatment day is indicated in the parenthesis. Expression on CD3+ CAR- cells is shown as reference. Cells gated on live CD3+ CD45+ cells. **b)** Percentage of PD1+ CD3+ CAR+ in the peripheral blood of patients at the peak of CAR marking that received the highest AUTO3 dose.

In terms of AUTO3 persistence, patients in cohort 2 (that is, at the AUTO3 dose of 3 × 10^6^ cells per kg) had a longer median duration of CAR T cell persistence (344 d; range, 63–539 d) than the patients who received 0.3–2 × 10^6^ cells per kg (median, 42 d; range, 20–257 d) or patients who received 4.3–5 × 10^6^ cells per kg (median, 28 d; range, 19–571 d) (Extended Data Fig. 9b). At the peak of expansion in the patients who received the highest dose, the expression of PD1 on CAR T cells was minimal, indicating that exhaustion was not the indicator for poor persistence in this cohort (Extended Data Fig. 10). Furthermore, post hoc analysis of the CAR T cell memory phenotype showed that patients in the highest dose cohort of 4.3–5 × 10^6^ cells per kg had a low level of CD4- and CD8-naive cells (median, 0.94% and 3.35%; range, 0.43–3.2% and 1.11–4.67%, respectively) (Extended Data Fig. 5), which might have resulted in the lack of persistency in this dose cohort.

Of note, longer CAR T cell persistence was observed in six patients who had infusion with products consisting of at least 5% CD4- and CD8-naive cells (median, 160 d; range, 20–539 d), with 3 of 6 patients having persistence lasting longer than 6 months. By contrast, only 1 of 6 patients who had received products with <5% CD4- and CD8-naive cells had persistence longer than 6 months. Overall, the persistence of AUTO3 in the responding patients was lower than that reported for tisagenlecleucel in the ELIANA study^1^ (median time to last detection in blood (T_last_) 119 d versus 168 d, respectively).

### Relapses and lack of CAR T cell persistence

**Fig. 5:**
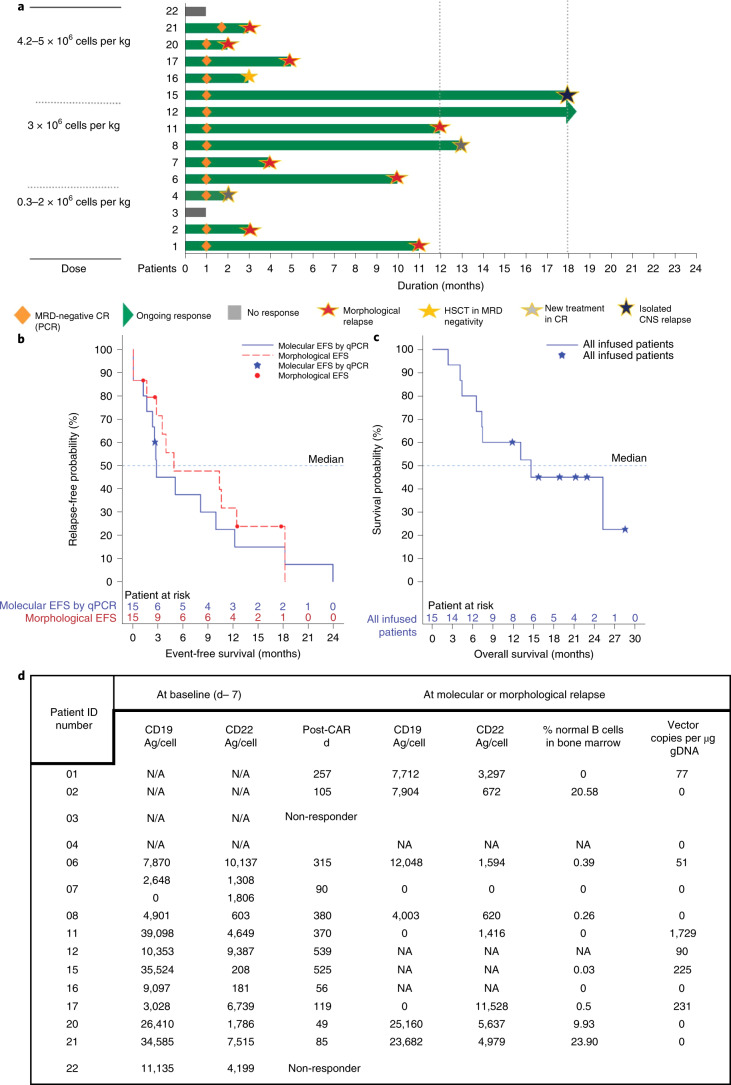
Clinical outcome and relapse phenotypes. **a**, Swimmer plot showing responses of individual patients infused with AUTO3, time to MRD negativity by qPCR and duration of response. CR, complete response. **b**, Kaplan–Meier curves of morphological and molecular event-free survival (EFS) in all patients (*n* = 15). Morphological EFS, non-response, morphological relapse, or death due to any cause (whichever occurs first); molecular EFS, non-response, morphological relapse, molecular relapse on qPCR (≥10^−4^) or death due to any cause (whichever occurs first). The blue filled stars and red filled circles represent censored observations. **c**, Kaplan–Meier curve of overall survival in all patients (*n* = 15). The blue filled stars represents censored observations. **d**, CD19 and CD22 expression density on the cell surface of leukemic blasts in bone marrow samples determined by flow cytometry at baseline (defined as d −7, the day before lymphodepletion) and at molecular or morphological relapse. Leukemic blasts were determined based on expression of CD45, CD3, CD19, CD20, CD22, CD10, CD34, CD73 and CD66. CD19 and CD22 density was calculated using antibodies bound per cell as a surrogate for antigens per cell (Ag/cell) using BD Quantibrite phycoerythrin beads as a reference. Blasts with mean fluorescence intensity (MFI) of CD19 or CD22 below twofold the MFI of CD19 or CD22 on T cells or on the samples stained with isotype controls were considered CD19 or CD22 negative. The percentage of normal B cells in the bone marrow samples was measured at the time of molecular or morphological relapse, when there was bone marrow sample available. Vector copy number per μg of genomic DNA in peripheral blood was measured around the time of molecular or morphological relapse, defined as the last time when CAR T cells were detectable on qPCR or the last assessment if zero copies were present. B cell recovery was defined as normal B cells ≥0.1% of the total lymphoid cells analyzed in bone marrow samples. Patient 07 had mixed CD19^−^ CD22^+^ and CD19^+^ CD22^+^ blast populations at baseline; antigen density analysis was performed separately for each population. Patient 15 relapsed with CNS disease and very low levels of bone marrow MRD (10^−5^ determined by qPCR). NA, data not available.

At 1 month after AUTO3 infusion 13 of 15 patients (86%) achieved complete response or complete response with incomplete bone marrow recovery. The rate of complete molecular remission (<10^−4^ as assessed with polymerase chain reaction (PCR) for leukemia-specific immunoglobulin heavy (IgH) gene rearrangements) was 80% (12 of 15 patients) at 1 month and 86% (13 of 15 patients) at 2 months. This included patient 7 who had a proportion of blasts that were CD19 negative prior to the start of the lymphodepletion. Two patients did not respond to the treatment (Fig. 5a). Of the 13 patients in complete response, nine have relapsed (of them, one patient had central nervous system (CNS) relapse with low levels of bone marrow MRD), three patients received new treatment for ALL while in complete remission (including one patient who underwent consolidative hemopoietic stem cell transplantation while in complete molecular remission) and one patient maintained complete molecular response without any additional treatment at 18 months after AUTO3 infusion (Fig. 5a).

CD19 loss was detected in blasts at relapse in three patients. This included patient 7 who had mixed CD19-positive and CD19-negative disease at study entry; notably CD22 was also lost in this patient upon relapse. Patient 11 also relapsed with CD19-negative disease with ongoing CAR T cell persistence > 1,000 copies per μg and B cell aplasia. CD22 antigen density was reduced in this patient upon relapse. In contrast, patient 17 relapsed with CD19-negative disease with low-level CAR T cell persistence and B cell recovery. In this case, there was no drop in CD22 density (Fig. 5d).

As of the cut-off date, 6 of 15 patients (40%) are alive. The overall survival rate was 80% at 6 months and 60% at 12 months for all treated patients, and 90% and 70%, respectively, for CAR-naive patients who received ≥3 × 10^6^ cells per kg AUTO3 (considered the active dose). The event-free survival rate, defined using the ELIANA study criteria^1^ as the time from AUTO3 infusion to no response or to morphological relapse or death (whichever occurred earlier), was 48% and 32% at 6 and 12 months, respectively (57% and 46%, respectively, in the CAR-naive group with active doses) (Fig. 5b,c). Using more stringent criteria^13^, under which an event is defined as molecular relapse or death, the molecular event-free survival rate was 38% and 23% at 6 and 12 months, respectively (58% and 35%, respectively, in the CAR-naive group with active doses) (Fig. 5b).

Due to the limited AUTO3 persistence at doses of up to 5 × 10^6^ cells per kg during the dose escalation phase in the pediatric and young adult cohorts and the lack of correlation between higher doses and CAR T cell persistence, the dose level of 10 × 10^6^ cells per kg was not tested and the adult cohort was not initiated.

## Discussion

In this study, we demonstrate the feasibility of dual targeting of CD19 and CD22 using a bicistronic CAR T cell therapy (AUTO3) in a cohort of pediatric and young adult patients with relapsed or refractory B cell ALL. Our data show no evidence of increased toxicity associated with dual antigen targeting despite 8 of 14 patients being treated in frank morphological relapse. Consistent with this, we found only modest elevations of IFN-γ, IL-6 and TNF-α in a minority of patients compared with the approved FMC63 CD19 CAR T cell therapy. Importantly, the safety profile was consistent in the patients receiving a single or split dose of AUTO3, independently of the marrow tumor burden or the clinical status of the patients. Although the small sample size of patients receiving single (*n* = 12 patients) and split dosing (*n* = 3 patients) makes any comparison between both dose schedules very difficult, our data do not suggest that an AUTO3 fractionated dose could have prevented the incidence of CAR T cell-related adverse events. Studies with a bigger sample size, however, are needed to confirm this.

Although the higher dose level of 10 × 10^6^ cells per kg was not tested, the 5 × 10^6^ cells per kg dose is similar to the maximum dose range approved for tisagenlecleucel in patients with relapsed or refractory B cell ALL with body weight ≤50 kg. And although our study used the Lee et al. scale^13^ for grading CRS whereas the ELIANA trial used the University of Pennsylvania scale^15^ (which make a comparison between the two trials difficult), if we apply the University of Pennsylvania scale in the AMELIA study only one patient treated with AUTO3 developed grade 3 CRS, whereas in the ELIANA study^1^ 16 and 19 of the 75 treated patients developed grade 3 and 4 CRS, respectively. Similarly, no patients developed grade 3 AUTO3-related neurotoxicity compared with 10 of the 75 treated patients in the ELIANA trial^1^, in which patients received a similar median CAR T cell dose of 3.1 × 10^6^ per kg (range, 0.2 × 10^6^–5.4 × 10^6^ cells per kg), suggesting the favorable safety profile of AUTO3.

Thirteen of 15 treated patients responded to the treatment, regardless of disease burden, cytogenetic risk factors or the number of prior lines of therapy. High complete molecular response rates, similar to those seen with tisagenlecleucel, were observed in all dose cohorts. Of note, patient 7, who had 5.5% CD19^−^CD22^+^ blasts before lymphodepletion, achieved molecular complete response, demonstrating the activity of the CD22 CAR.

Nine patients ultimately relapsed. Of these, eight had low CAR T cells at relapse (<1,000 copies per μg) and five had detectable normal B cells in marrow aspirates. This suggests that the predominant cause of treatment failure was lack of sufficient long-term CAR T cell persistence. However, indications of CAR T cell modulation of the target antigen were observed. For instance, one patient who had mixed CD19-positive and -negative disease at treatment relapsed with CD19- and CD22-double negative disease, suggesting that the CD19-negative subclone outgrew and then modulated CD22 expression. A further patient relapsed despite having >1,000 CAR T copies per μg and B cell aplasia. This patient relapsed with CD19-negative disease and dimming of CD22 expression, as previously described after CD22 CAR T cell therapy^3,4^. It is thus possible that optimization of CD22 CAR recognition of low-antigen-density targets may be required to improve the efficacy of bispecific CARs in future studies. One further patient relapsed with CD19-negative disease without dimming of CD22 expression. In that case, CAR T persistence was low with return of normal B cells, perhaps indicating that an evolving antigen escape had been cut short by CAR T cell loss.

The reason for the reduced persistence of AUTO3 in our study is not fully established. Immune rejection is unlikely given the humanization of binding domains and the profound B cell aplasia observed after CAR T cell treatment. Also, in silico analysis predicted reduced immunogenicity when compared with the binding domain used in tisagenlecleucel (Extended Data Fig. 1c). The inability to detect CAR T cells on flow cytometry at later time points prevents assessment of whether dual signaling led to CAR T cell exhaustion. However, the complete loss of CAR T cells seen on quantitative PCR (qPCR) in 4 of 9 patients argues against this, and the infused products did not have an exhausted phenotype. We believe that the limited persistence of AUTO3 in vivo probably reflects the differentiated phenotype of the product in most patients (Extended Data Fig. 5), which may in turn reflect the manufacturing methodology used. Data from both murine studies^16^ and integration site analysis after adoptive T cell transfer in humans^17^ suggest that long-term persisting T cells are derived predominantly from the stem cell-like memory T cell (T_SCM_) and central memory T cell (T_CM_) compartments of the infused product. Our own data suggest that this is also likely to be true after CAR T cell therapy^18^.

The lack of long-term CAR T cell persistence has also been observed in other approaches involving dual CD19 and CD22 antigen targeting with CARs. In the Wang et al.^5^ study in which a cocktail of CD19 and CD22 CAR T cells were co-administered, persistence of both CAR T cell populations was short (median time to recovery of B cell hematogones, 4 months) and, consistent with this, 23 of 24 relapses were with CD19^+^ CD22^+^ disease, resulting in a 1 year progression-free survival rate (52.9%) similar to that seen with tisagenlecleucel alone^19^. Likewise, early data from the Seattle Pediatric and Young Adult Leukemia Adoptive Therapy (PLAT)-05 study^20^ of T cells co-transduced with lentiviral vectors encoding CD19 and CD22 CARs and the Stanford–National Cancer Institute study^7^ using a CD19–C22 tandem CAR suggest that limited CAR T cell persistence makes the assessment of the effect of dual targeting on antigen-negative relapse challenging. Interestingly, in another phase 1 clinical trial^21^ with a bispecific CAR capable of simultaneously recognizing CD22 and CD19 in adult patients with relapsed or refractory B-ALL and large B cell lymphoma, in contrast to our study, none of the relapses was associated with CD22 antigen loss or low expression, suggesting that potency toward the CD22 antigen was insufficient in this other dual targeting CAR construct. Taken together, these data highlight the importance of enhancing potency against the CD22 antigen and of CAR T cell persistence to maximize patient benefit from dual targeting strategies. A number of potential approaches are being studied to achieve this, including shortening the duration of manufacture^22^ and incorporating small molecules such as AKT inhibitors^23^ to favor retention of an early T_SCM_–T_CM_ phenotype.

In conclusion, we demonstrate the feasibility and safety of dual targeting of CD19 and CD22 in ALL using bicistronic CAR T cells. Although the complete molecular response of patient 7 who had CD19-negative disease prior to CAR T cell therapy and the emergence of CD22-negative or -dim relapse in some patients confirms the activity of the CD22 CAR, the limited persistence of AUTO3 precludes assessment of the impact of dual targeting on relapse rates. AUTO3 did not seem to be superior to the approved CD19 CAR T cell therapy in pediatric and young adult patients with relapsed or refractory B cell ALL. Dual CD19 and CD22 targeting resulted in a 1 year overall survival rate of 60% and an event-free survival rate of 32%, indicating a substantial clinical benefit of AUTO3 therapy in the relapsed or refractory B cell ALL setting. Nevertheless, strategies to further improve CAR T cell persistence and to further target leukemic clones expressing low levels of CD22 are needed to fully realize the potential of dual targeting CAR T cell therapy in B cell ALL.

## Methods

### Antibody humanization

The antibody HD37 was produced from a hybridoma clone generated through the fusion of spleen cells from BALB/c mice immunized with cells from a patient with hairy cell leukemia and NS1-Ag4/1 myeloma cells^24^. LT22 was produced from a hybridoma clone derived through the fusion of spleen cells from BALB/c mice immunized with the Daudi cell line and Sp2/0 myeloma cells. Antibody sequences derived from the LT22 and HD37 hybridomas were used as the source of complementarity-determining regions (CDRs) for framework grafting. CDRs of HD37 and LT22 were defined based on the Kabat numbering system^25^ and inserted in silico into the human frameworks selected for humanized heavy and light chains. Structural homology models were generated by comparing the sequences of HD37 and LT22 to all antibody sequences in the Protein Data Bank database. Antibody modeling and refinement was carried out using BioLuminate with PRIME (Schrodinger)^26^. The quality of the homology models was verified by Ramachandran analysis of the final energy-minimized structures. The homology models were used as the basis for CDR grafting based on the crystal structure database of human antibodies. From a database search the best weighted scores from verified human antibodies were selected to provide framework regions of humanized antibodies.

### Antibody expression

Protein expression was carried out in 250 ml baffled flasks with a 30 ml volume of Expression medium in each flask. Flasks were agitated at a speed of 125 r.p.m. at 37 °C under 8% CO_2_. For each 30 ml transfection, 7.5 × 10^6^ cells were seeded in 25.5 ml Expi293 Expression medium. On the day of transfection the transfection volume was made up to 30 ml, the number of cells adjusted to 2.5 × 10^6^ cells per ml, and cultures of >95% viability were used. A total of 30 μg DNA was used for transfection, and 1 μg DNA per ml transfection reaction was mixed with 2.7 μl ExpiFectamine 293. The transfection mix was incubated for 10 min before being applied to the cells.

### Antibody surface plasmon resonance

Recombinant CD19 or CD22 at known concentrations was supplemented with BSA to a final concentration of 0.1% prior to overnight dialysis against HBSP+ buffer (Cytiva). The HBSP+ buffer used for dialysis was filtered to 0.2 μm and used as the running buffer in BIAcore experiments. Sensograms were obtained by capturing an Fc-tagged single-chain variable fragment (scFv) or monoclonal antibody (ligand) on the surface of a CM5 chip pre-immobilized with anti-mouse antibody (Cytiva) or a protein A chip, to a density of 100–300 RU. Recombinant, purified CD19 or CD22 was used as the analyte and injected over the respective flow cells. In each experiment, flow cell 1 was unmodified and was used for reference subtraction. A 0 concentration sensogram of buffer alone was used as a double reference subtraction to factor for drift. Data were fitted to a 1:1 Langmuir binding model. Given that a capture system was used, a local R_max_ parameter was used for the data fitting in each case^27^. For competition binding assessment, purified recombinant FMC63 was covalently immobilized on a CM5 chip to 3,000 RU. Two analytes were injected at flow rates of 30 µl per min sequentially: the first contained recombinant CD19 with a C-terminal polyhis tag, while the second contained either a recombinant purified anti-CD19 scFv or an anti-His monoclonal antibody (Cytiva). Independent cycles were carried out using FMC63, HD37 and the anti-His antibody as the second analyte. Regeneration was carried out with 2.5 M glycine, pH 3.0, between each cycle.

### Immunogenicity prediction

The T20 score analyzer was used to calculate the humanness scores from the CAR binding domains compared with germline human antibody variable sequences^28^. The sequences of the grafted antibody frameworks were analyzed through the T20 score analyzer (https://dm.lakepharma.com/bioinformatics/). A score of 85 or more is associated to a human sequence. To determine human major histocompatibility complex (MHC) class I affinity, the sequence of the grafted antibody frameworks was analyzed using the Immune Epitope Database and Analysis Resource (IEDB) proteasomal cleavage–TAP transport–MHC class I combined predictor (http://tools.iedb.org/processing/) on the human HLA (human leukocyte antigen) allele A*02:01 using all peptide length (8–14) (ref. ^29^).

### In vitro functional experimentation

Transduced T cells were depleted of CD56-positive cells prior to co-culture with target cells. Target cells were either Raji wild type (WT), CRISPR-Cas9 engineered CD19 knockout (CD19KO) Raji cells or SupT1 cells transduced to express CD19 or CD22. Cytotoxicity was assessed by flow cytometry and normalized to that of non-transduced T cells. Remaining viable target cells were defined by their exclusion of 7-amino-actinomycin D and the absence of expression of CD3. Cytokine concentration was measured using ELISA MAX Deluxe Set Human IFN-γ (BioLegend; 430104) according to the manufacturer’s instructions.

### In vivo functional experimentation

All animal work was performed with the approval of the local ethics committee and in compliance with United Kingdom Home Office requirements. The 6–8-week-old female NSG mice were injected intravenously with WT Nalm6 or CD19KO Nalm6 cells that were transduced with luciferase. Tumor engraftment was assessed using bioluminescent imaging and mice were stratified into groups. After a period of tumor cell engraftment (4 d for WT and 8 d for CD19KO Nalm6), the mice were given 5 × 10^6^ transduced CAR T cells or a matched number of non-transduced T cells. Mice injected with WT and CD19KO Nalm6 were killed 14 and 15 d, respectively, after the administration of T cells.

### Gene analysis

CAR T cells were sorted using anti-CD34 magnetic beads (Miltenyi) and activated for 24 h with 50 ng per ml CAR-specific anti-idiotype. After stimulation, RNA was extracted from CAR T cells using the Quick RNA kit (Zymo Research). Samples were prepared according to the manufacturer’s protocols for the Nanostring nCounter CAR-T Characterization Panel (Nanostring). A list of genes and target probe sequences can be found at www.nanostring.com. Cartridges were run on the nCounter Sprint Profiler. Data analysis was conducted using the nSolver software and R studio v3.6.

### Vector production

Vector was produced by lipofectamine-facilitated transient transfection of 293T cells with plasmids encoding for Moloney murine leukemia virus gag–pol, the RD114 envelope and the transfer vector. Supernatant was subjected to purification by anion exchange chromatography.

### CAR T cell production

CAR T cell production was performed on the Prodigy device (Miltenyi). Pheresis was washed in DPBS, stimulated with transact (Miltenyi) and cultured in IL-7 and IL-15. Transduction was performed on d 3 after stimulation initially in retronectin-coated bags, but subsequently was performed in suspension within the Miltenyi Prodigy bioreactor facilitated by vectofusin (Miltenyi). T cells were expanded in the Prodigy device until the dose was reached. Total production time ranged from 7 to 14 d. Products were cryopreserved in DMSO containing cryoprotectant.

### Immunophenotyping of cell products

Cryopreserved leukapheresates and cell products were analyzed using flow cytometry. Transduced cells were identified with an anti-idiotype antibody specific for the HD37 anti-CD19 binder. Memory and exhaustion phenotypes in CD4-positive and CD8-positive CAR T cells were based on the expression of CD45RA, CD45RO, CD62L, CCR7, PD-1, LAG-3, TIM-3 and TIGIT. The list of antibodies used and an example of the gating hierarchy are given in the Supplementary Information.

### Study design

The original design of the single-arm AMELIA trial consisted of two parts, a phase 1 dose escalation phase and a phase 2 expansion phase. The phase 1 portion of the study was originally planned to evaluate four doses of AUTO3 in pediatric and young adult patients with relapsed or refractory B cell ALL (aged 1–24 years), starting at 1.0 × 10^6^ CAR T cells per kg (cohort 1), 3 × 10^6^ CAR T cells per kg (cohort 2), 5×10^6^ CAR T cells per kg (cohort 3) or 10 × 10^6^ CAR T cells per kg (cohort 4) as a single or split dose based on disease burden. Patients with <25% bone marrow blasts on the day prior to the initiation of the lymphodepletion received AUTO3 as a single dose and those with ≥25% bone marrow blasts received AUTO3 as a split dose The study design also included an optional adult B cell ALL cohort (≥25 years) at the highest pediatric or young adult dose that has been declared safe. Patients should receive pre-conditioning with 30 mg m^−2^ d^−1^ fludarabine for 4 d and 500 mg m^−2^ d^−1^ cyclophosphamide for 2 d prior to AUTO3 infusion.

Dose escalation followed a rolling six design^30^. Each cohort could treat up to six patients. Evaluation of a dose level required at least three patients treated at the planned dose level to have completed the 30 d DLT evaluation period to declare any dose cohort and schedule safe.

The study was approved by the UK Medicines and Healthcare Products Regulatory Agency (clinical trial authorization no. 46113/0002/001), the London–West London and GTAC (Gene Therapy Advisory Committee) Research Ethics Committee (REC ref. no. 17/LO/0506), and the research and development department of each participating National Health Service trust. Autolus Therapeutics sponsored this clinical study. The trial was prospectively registered and entered in the EUDRA CT database on 3 March 2017 (EUDRA CT 2016-004680-39). The study was posted at ClinicalTrials.gov on 21 September 2017 (identifier NCT03289455). The study was conducted in three hospitals: Great Ormond Street Hospital for Children, London, UK, University College London Hospitals NHS Foundation Trust, London, UK and Royal Manchester Children’s Hospital, Manchester, UK, and all patients provided consent according to the local ethics committees requirements. No compensation schemes were implemented during the duration of this trial.

### Primary endpoints

These consisted of the incidence of grade 3–5 toxicity during the DLT period (that is, 30 d after the last dose of AUTO3), and the frequency of DLT of AUTO3.

### Secondary endpoints

The secondary endpoints were the frequency and severity of adverse events and serious adverse events, and the proportion of patients for whom an AUTO3 product can be generated (feasibility); the proportion of patients (all and prior CD19 CAR T treatment naive) achieving morphological remission (complete response or complete response with incomplete bone marrow recovery) and/or MRD-negative complete response in bone marrow within 30 d (±3 d) after the first AUTO3 infusion on qPCR and/or flow cytometry; relapse-free survival; event-free survival; overall survival; the proportion of patients in molecular remission without further therapy at 6 months and at 1 and 2 years following treatment with AUTO3; the incidence of CD19- and/or CD22-negative relapse; the measurement of CAR T cells levels on qPCR and/or flow cytometry at a range of time points in the peripheral blood and bone marrow; and the depletion of circulating B cells assessed on flow cytometry at a range of time points.

### Dose-limiting toxicities

The DLT evaluation period was 30 d (±3 d) after the last dose of AUTO3. DLTs were defined as any new non-hematological adverse event of grade 3 or higher toxicity using the National Cancer Institute Common Terminology Criteria for Adverse Events (CTCAE) v5.0, which is probably or definitely related to AUTO3 therapy, which occurs within the DLT evaluation period, and which fails to resolve to grade 2 or better within 14 d, despite appropriate supportive measures; a grade 4 CRS or neurotoxicity, cerebral edema, or grade 3 neurotoxicity (including cerebral edema) that lasts >72 h; grade >3 disseminated intravascular coagulation; grade >2 infusion reaction; or any other fatal event (grade 5) or life-threatening event (grade 4) that cannot be managed with conventional supportive measures or which in the opinion of the safety evaluation committee (SEC) necessitates dose reduction or other modification to trial treatment to avoid a similar hazard in future patients.

### Safety evaluation committees

Patient safety was monitored throughout all parts of the study by an SEC established by the Sponsor. The SEC consisted of the principal investigators and Sponsor staff. The SEC monitored treatment-emergent data on an ongoing basis throughout study conduct for the purpose of ensuring the continued safety of patients enrolled in this study. During phase 1 (dose escalation), the SEC were scheduled to meet after the first patient in each cohort completed 2 weeks; after the third patient in each cohort completed the DLT evaluation period of 30 d ±3 d after the last dose of AUTO3 in the case of split dosing; in additional ad hoc meetings if safety stopping criteria were met; and when clinically necessary based on emerging data.

An independent data monitoring committee (IDMC), consisting of two independent physicians and one statistician, was established by the Sponsor to review serious safety events. The IDMC were scheduled to meet when any safety stopping criteria were met.

### Inclusion and exclusion criteria

Eligible patients were male or female children and young adults (age 1–24 years) with relapsed or refractory B-lineage ALL.

#### Inclusion criteria

The inclusion criteria are as follows. Male or female sex, age 1–24 years, high-risk relapsed or refractory B-lineage ALL and: any bone marrow relapse or CNS relapse with detectable bone marrow disease (>10^−4^) on flow cytometry or molecular MRD after allogeneic stem cell transplantation (SCT) and ≥6 months from SCT at the time of AUTO3 infusion; or high-risk first relapse (as per International Study for Treatment of Childhood Relapsed ALL criteria); or standard risk relapse with HR cytogenetics (HR defined as mixed linkage leukemia gene rearrangement (KMT2A), intrachromosomal amplification of chromosome 21 amplification, near-triploidy (60–78 chromosomes) or near-haploidy; or second or greater relapse; or bone marrow MRD ≥ 10^−3^ prior to planned SCT; or any on-treatment relapse in patients aged 16–24 years. Documentation of CD19 and or CD22 expression on leukemic blasts in the bone marrow, peripheral blood, or cerebrospinal fluid on flow cytometry within 3 months of screening. Detectable disease in the bone marrow at a level ≥10^−4^ using molecular- or flow cytometry-based methods (phase 1 only) at enrollment (patients developing ≤10^−4^ bone marrow disease due to bridging therapy may continue to receive AUTO3). Absolute lymphocyte count ≥0.5 × 10^9^ per liter at enrollment. Adequate renal, hepatic, pulmonary and cardiac function defined as serum creatinine based on age and gender ≤1.5-fold the upper limit of normal, serum alanine aminotransferase and aspartate aminotransferase ≤5-fold the upper limit of normal, total bilirubin ≤2-fold the upper limit of normal, except in patients with Gilbert’s syndrome, left ventricular shortening fraction ≥28% confirmed on echocardiography or left ventricular ejection fraction ≥45% confirmed on echocardiography, and baseline oxygen saturation >92% on room air. Karnofsky (age 10–24 years) or Lansky (age < 10 years) score ≥50%. Willing and able to give written, informed consent to the current study (patient and/or parent or legal guardian).

#### Exclusion criteria

The exclusion criteria are given as follows. Isolated extramedullary disease relapse (in phase 2 of the study, patients with isolated CNS relapse after SCT or before SCT if high risk, aged 16–24 years on therapy relapse or second relapse or greater, with ≤CNS grade 2 disease at the time of enrollment are eligible). Active CNS involvement of ALL, defined as CNS grade 3 as per the National Comprehensive Cancer Network guidelines. Patients developing CNS grade 3 disease at any time after enrollment will also be excluded. Active infectious bacterial or viral disease (hepatitis B virus, hepatitis C virus, human immunodeficiency virus, human T cell lymphotropic virus, syphilis, West Nile (United States only) or Zika viruses (United States only)) requiring intravenous anti-microbials for treatment. Pregnancy or lactation in female patients. Child-bearing potential in female patients (defined as the physiological capability of becoming pregnant) and the unwillingness, in male patients, to use highly effective methods of contraception during the treatment period and for a period of 1 year after the AUTO3 infusion. Inability to tolerate leukapheresis. Prior CD19 or CD22 targeted therapy with grade 4 toxicity (except for hematological toxicity) or ≥grade 3 CRS or ≥grade 3 drug-related CNS toxicity. CD22 targeted therapy such as inotuzumab ozogamicin in patients who are CD19 negative (unless it is demonstrated that this therapy had no effect on CD22 target expression). Pre-existing significant neurological disorder (other than CNS involvement of underlying hematological malignancy). In SCT patients only: active significant (overall grade ≥ II, Seattle criteria) acute graft-versus-host disease (GVHD) or moderate–severe chronic GVHD (National Institutes of Health consensus criteria) requiring systemic steroids or other immunosuppressants within 4 weeks after enrollment. The following medications are excluded: steroids (therapeutic doses of steroids must be stopped >72 h prior to AUTO3 infusion and leukapheresis, however, physiological replacement doses of steroids are allowed: <12 mg m^−2^ d^−1^ hydrocortisone or equivalent); allogeneic cellular therapy (any donor lymphocyte infusions must be completed >6 weeks prior to AUTO3 infusion); GVHD therapies (any drug used for GVHD must be stopped >4 weeks prior to AUTO3 infusion); chemotherapy (should be stopped 1 week prior to leukapheresis and 2 d prior to starting pre-conditioning chemotherapy); intrathecal therapy (methotrexate within 4 weeks and other intrathecal chemotherapy (for example, cytarabine) within 2 weeks prior to starting pre-conditioning chemotherapy); and live vaccine ≤4 weeks prior to enrollment. Known allergy to albumin, DMSO, cyclophosphamide or fludarabine. Any other condition that in the Investigator’s opinion would prevent the patient from undergoing protocol-based therapy.

### Toxicity assessment

Adverse events were graded according to CTCAE v4.03. CRS was graded according to the Lee et al. criteria^13^ and neurotoxicity as per the ASBMT (American Society for Blood and Marrow Transplantation) guidelines for immune effector cell-associated neurotoxicity syndrome (ICANS)^13^. Laboratory safety assessments were based on blood samples collected during and after AUTO3 infusion. Samples were analyzed locally for complete blood counts, biochemical assays, renal function, hepatic function, coagulation and serum immunoglobulin concentrations.

### Response assessment

Response evaluations for the primary endpoint and final analysis were based on the response criteria for ALL according to the National Comprehensive Cancer Network guidelines v2 (2014).

### Serum cytokines

Cryopreserved serum cytokine samples were assessed at screening (d −84 to d −35), d −6, 0, 2, 5, 7, 9, 12, 14, 21, 28 and month 3 after CAR T cell infusion using sandwich immunoassay multiplex analysis of IL-2, IL-5, IL-6, IL-7, IL-8, IL-10, IL-15, granulocyte–macrophage colony-stimulating factor, TNF-α and IFN-γ (Meso Scale Diagnostics) according to the manufacturer’s instructions. The validated lower limit of this assay for the different cytokines ranges between 0.2 and 1.5 pg per ml.

### qPCR analysis of CAR T cell expansion and persistence

To assess CAR T cell expansion and persistence, 9–18 ml peripheral blood was collected in lithium heparin tubes and 2.5 ml in PAXgene tubes on d 2, 5, 7, 9, 12, 14, 21, 30 and months 2, 3, 4, 6, 8, 10, 12, 15, 18, 21, 24.

CAR T cells were detected using a validated qPCR assay detecting the following transgene-specific sequences targeting the retroviral Psi sequence in the vector: Psi forward primer, GTGTCTGTCCGATTGTCTAGTG; Psi reverse primer, CCGCCAGATACAGAGCTAGTTA; and Psi FAM probe, TTTATGCGCCTGCGTCGGTACTAG. Genomic DNA was isolated and reactions carried out with retroviral PSI specific primers and PrimeTime probes (Integrated DNA Technologies), using a minimum of 95 ng genomic DNA. Primers specific for the albumin gene were used as internal controls. Results were reported as copies of the transgene per µg genomic DNA, with a detection limit of 95 copies per µg DNA. To achieve higher sensitivity and better accuracy, the assay was transferred to droplet digital PCR technology using the following Psi specific primers (Integrated DNA Technologies): Psi forward primer, TGGCCAGCAACTTATCTGTG; Psi reverse primer, TTCCGAACTCGTCAGTTCCA; and Psi FAM probe, ACTAGTACC GACGCAGGCGCA. RPP30 was used as an internal control. Reactions were performed with 0.1 µg genomic DNA. The limit of detection of this assay was 54.4 copies per µg DNA.

### Analysis of cellular kinetics

CAR T cell kinetics were analyzed by measuring the AUC up to d 28 or d 84 (AUC_d0–28_ and AUC_d0–84_) using a trapezoidal algorithm. C_max_ was the peak concentration of CAR T cells documented, T_max_ was the time in days from infusion to maximum CAR T cell concentration and T_last_ was the time from infusion to the last documented detection of CAR T cells. T_1/2_ was the half-life of CAR T cell persistence over the contraction phase, as measured in patients with a minimum of three data points documented after T_max_.

### Minimal residual disease

MRD was determined using a nationally accredited qPCR assay for leukemia-specific IgH gene rearrangements, in the Bristol Genetics Laboratory (CPA reference no. 2907). A sample was determined to be MRD positive when the number of amplification cycles required to reach a fixed signal threshold (Ct) in at least one of three replicates was ≤4.0 Ct from the point representing the sensitivity and was not >20 cycles from the intercept of the standard curve. The sensitivity of the assay was 10^−4^ (0.01%).

### CD19 and CD22 status at molecular or morphological relapse

Bone marrow aspirate samples at molecular or morphological relapse were analyzed using flow cytometry in the Bristol Genetics Laboratory. CD19 and CD22 expression density on the cell surface of leukemic blasts was measured at baseline (d −7, the day before lymphodepletion) and at molecular or morphological relapse using BD Quantibrite phycoerythrin beads. Leukemic blasts were identified based on the expression of CD45, CD3, CD19, CD20, CD22, CD10, CD34, CD73 and CD66. Blasts with mean fluorescence intensity (MFI) of CD19 or CD22 below twofold the MFI of CD19 or CD22 on T cells or on control samples stained with isotype controls were considered CD19 or CD22 negative. A list of antibodies and an example of the gating hierarchy can be found in the Supplementary Information.

### Quantification of bone marrow B cells

B cell counts from bone marrow samples were quantified using multiparameter flow cytometry. A total of at least 500 lymphoid events was needed to consider a bone marrow aspirate sample evaluable for the assessment of B cell aplasia and B cell recovery. Lymphocytes were identified in forward scatter versus side scatter plots and the expression of specific B cell markers (CD19 and CD22) was used to calculate the percentage of normal B cells (including hematogones). A separate gating strategy for leukemic B cell blasts counts was added to exclude the leukemic blasts from the total normal B cell counts. B cell bone marrow recovery was defined as ≥0.1% total normal B cells in the total lymphoid cells analyzed in bone marrow samples.

### Statistical analysis

GraphPad Prism 8 was used for all pre-clinical data analysis, utilizing two-tailed statistical tests with the assumption of normality. The application of two-way analysis of variance with Bonferroni correction was used to account for time and the different CAR cohorts. Flow cytometry data were analyzed using FlowJo v10.1.1, FCS Express v7.06.0015 and BD FACSuite v1.3. Transcriptomics data were analyzed using nSolver v2.0.134 and R studio 3.6. Clinical data are captured in the clinical database via the Encapsia electronic data capture (EDC) system v1.0. SAS 9.4 was used for clinical data analysis. Categorical variables are reported in terms of frequency and percentage, and continuous variables in terms of median and range unless otherwise specified. Time-to-event outcomes were summarized using the Kaplan–Meier method. Toxicity events are reported at the maximum grade experienced according to the CTCAE. The cellular kinetics parameters were estimated from the individual concentration versus time profiles using a non-compartmental approach in Phoenix WinNonlin.

### Reporting Summary

Further information on research design is available in the Nature Research Reporting Summary linked to this article.

## Online content

Any methods, additional references, Nature Research reporting summaries, source data, extended data, supplementary information, acknowledgements, peer review information; details of author contributions and competing interests; and statements of data and code availability are available at 10.1038/s41591-021-01497-1.

## Supplementary information

Supplementary Information Supplementary Table 1, List of antibodies, catalog numbers and validation. Supplementary Figure 1, Representative example of gating hierarchy used for the analysis of memory and exhaustion phenotypes in CAR T cell products. Supplementary Figure 2, Representative example of gating hierarchy used for the analysis of leukemic blasts in bone marrow.

Reporting Summary

## Data Availability

The raw pre-clinical and clinical datasets generated during and analyzed in the current study are not publicly available due to proprietary ownership. All requests for raw and analyzed data and materials should be addressed to the corresponding author and will be reviewed by Autolus PLC to verify whether the request is subject to any intellectual property or confidentiality obligations. Patient data may be subject to patient confidentiality. Any data and materials that can be shared will be released via a material transfer agreement.

## Extended data

are available for this paper at 10.1038/s41591-021-01497-1.
