# Supplementary material for: CAR T cells with dual targeting of CD19 and CD22 in pediatric and young adult patients with relapsed or refractory B cell acute lymphoblastic leukemia: a phase 1 trial
Source: Nat Med. 2021 Oct 12;27(10):1797–805. doi: 10.1038/s41591-021-01497-1 (PMC8516648; doi:10.1038/s41591-021-01497-1)
Supplement: Supplementary file 1 — Supplementary Table 1, List of antibodies, catalog numbers and validation. Supplementary Figure 1, Representative example of gating hierarchy used for the analysis of memory and exhaustion phenotypes in CAR T cell products. Supplementary Figure 2, Representative example of gating hierarchy used for the analysis of leukemic blasts in bone marrow. [file 41591_2021_1497_MOESM1_ESM.pdf]

nature portfolio

https://doi.org/10.1038/s41591-021-01497-1

Supplementary information

CAR T cells with dual targeting of CD19 and CD22 in pediatric and young adult patients with relapsed or refractory B cell acute lymphoblastic leukemia: a phase 1 trial

In the format provided by the authors and unedited

Supplementary Table 1

Antigen	Fluorochrome	Clone	Vol./test or dilution	Supplier	Cat.#	Validation information
CCR7	PE-Dazzle	G043H7	2.5 µL	BioLegend	353236	https://www.biolegend.com/en-us/products/pe-dazzle-594-anti-human-cd197-ccr7-antibody-9811?GroupID=BLG9611
CD10	BV786	HI10a	2.5 µL	BD Biosciences	564960	https://www.bdbiosciences.com/en-ca/products/reagents/flow-cytometry-reagents/research-reagents/single-color-antibodies-ruo/bv786-mouse-anti-human-cd10.564960
CD123	BV421	6H6	1 µL	BioLegend	306018	https://www.biolegend.com/en-us/products/brilliant-violet-421-anti-human-cd123-antibody-7277
CD19	PE	SJ25C1	0.6 µL	BD Biosciences	345789	https://www.bdbiosciences.com/ds/europe/tds/23-5061.pdf
CD19	APC	SJ25C1	0.6 µL	BD Biosciences	345791	https://www.bdbiosciences.com/sg/reagents/clinical/reagents/single-antibodies/cd19-apc-sj25c1-also-known-as-sj25-c1/p/345791
CD20	APC-H7	L27	2.5 µL	BD Biosciences	641396	https://www.bdbiosciences.com/us/applications/research/stem-cell-research/hematopoietic-stem-cell-markers/human/negative-markers/apc-h7-mouse-anti-human-cd20-l27/p/641396
CD22	PE	S-HCL-1	1.2 µL	BD Biosciences	337899	https://www.bdbiosciences.com/ds/europe/tds/23-6995.pdf
CD27	BV711	M-T271	0.31 µL	BD Biosciences	564893	https://www.bdbiosciences.com/us/p/564893
CD28	PerCP Cy5.5	CD28.2	1.25 µL	BioLegend	302922	https://www.biolegend.com/en-us/products/percp-cyanine55-anti-human-cd28-antibody-4234?GroupID=BLG10175
CD3	APC R700	SK7	1.25 µL	BD Biosciences	659119	https://www.bdbiosciences.com/eu/p/659119
CD3	PE.Cy7	SK7	0.3 µL	BioLegend	344816	https://www.biolegend.com/en-us/products/pe-cyanine7-anti-human-cd3-antibody-6934?GroupID=BLG5900
CD34	APC-R700	8G12	2.5 µL	BD Biosciences	659123	https://www.bdbiosciences.com/en-ca/products/reagents/flow-cytometry-reagents/clinical-discovery-research/single-color-antibodies-ruo-gmp/apc-r700-mouse-anti-human-cd34.659123
CD4	BUV805	SK3	0.32 µL	BD Biosciences	612887	https://www.bdbiosciences.com/in/reagents/research/antibodies-buffers/immunology-reagents/anti-human-antibodies/cell-surface-antigens/buv805-mouse-anti-human-cd4-sk3-also-known-as-leu3a/p/612887
CD4	BUV496	SK3	1 µL	BD Biosciences	564651	https://www.bdbiosciences.com/ds/pm/tds/564651.pdf
CD45	BV711	HI30	2.5 µL	BioLegend	304050	https://www.biolegend.com/en-us/search-results/brilliant-violet-711-anti-human-cd45-antibody-9736
CD45RA	BV480	HI100	0.16 µL	BD Biosciences	566114	https://www.bdbiosciences.com/eu/applications/research/b-cell-research/surface-markers/human/bv480-mouse-anti-human-cd45ra-hi100/p/566155
CD45RO	BV605	UCHL1	0.62 µL	BD Biosciences	562791	https://www.bdbiosciences.com/eu/p/562791
CD62L	BV650	DREG56	0.31 µL	BD Biosciences	563808	https://www.bdbiosciences.com/us/p/563808
CD66c	FITC	KOR-SA3544	2.5 µL	Beckman Coulter	IM2039U	https://www.mybeckman.uk/reagents/coulter-flow-cytometry/antibodies-and-kits/single-color-antibodies/cd66c/im2039u
CD73	BV605	AD2	0.3 µL	BD Biosciences	563199	https://www.bdbiosciences.com/en-ca/products/reagents/flow-cytometry-reagents/research-reagents/single-color-antibodies-ruo/bv605-mouse-anti-human-cd73.563199
CD8	v500	RPA-T8	0.08 µL	BD Biosciences	560774	https://www.bdbiosciences.com/en-us/products/reagents/flow-cytometry-reagents/research-reagents/single-color-antibodies-ruo/v500-mouse-anti-human-cd8.560775
CD8	BV786	RPA-T8	1.25 µL	BD Biosciences	563823	http://www.bdbiosciences.com/us/reagents/research/antibodies-buffers/immunology-reagents/anti-human-antibodies/cell-surface-antigens/bv786-mouse-anti-human-cd8-rpa-t8/p/563823
CD95	BUV395	DX2	0.16 µL	BD Biosciences	740306	https://www.bdbiosciences.com/en-us/products/reagents/flow-cytometry-reagents/research-reagents/single-color-antibodies-ruo/buv395-mouse-anti-human-cd95.740306
HLA-DR	BV510	L243	0.6 µL	BioLegend	307646	https://www.biolegend.com/it-it/products/brilliant-violet-510-anti-human-hla-dr-antibody-8008
LAG3	FITC	11C3C65	5 µL	BioLegend	369308	https://www.biolegend.com/en-us/products/fitc-anti-human-cd223-lag-3-antibody-13550?GroupID=BLG14890
PD-1	BUV737	EH12.1	2 µL	BD Biosciences	612791	https://www.bdbiosciences.com/en-us/products/reagents/flow-cytometry-reagents/research-reagents/single-color-antibodies-ruo/buv737-mouse-anti-human-cd279-pd-1.612791
TIGIT	PerCP Cy5.5	MBSA43	5 µL	Thermo Fisher	46-9500-42	https://www.thermofisher.com/antibody/product/TIGIT-Antibody-clone-MBSA43-Monoclonal/46-9500-42
TIM3	BV711	7D3	0.16 µL	BD Biosciences	565566	https://www.bdbiosciences.com/eu/applications/research/t-cell-immunology/th-2-cells/surface-markers/human/bv711-mouse-anti-human-tim-3-cd366-7d3/p/565566
Fixable viability dye	FVS780	-	1:1000	BD Biosciences	565388	https://www.bdbiosciences.com/en-us/products/reagents/flow-cytometry-reagents/research-reagents/single-color-antibodies-ruo/fixable-viability-stain-780.565388
CD22-Fc-biotin	-	-	10 µg/mL	Avitag Acro Biosystems	SI2-H82F8	https://www.acrobiosystems.com/P2524-Biotinylated-Human-Siglec-2--CD22-Protein-FcAvitag%E2%84%A2-%28MALS-verified%29.html
HD37 anti-idiotype	PE	-	2.5 µL	In house	-	Anti-idiotype antibody to HuHD37 was generated via murine immunisation and hybridoma screening. Binding specificity for HuHD37 CAR was assessed in comparison to non-relevant CARs
Streptavidin	AF647	-	1:50	BioLegend	405237	https://www.biolegend.com/en-gb/products/alexa-fluor-647-streptavidin-9305?GroupID=GROUP23
anti-His	-	H3	1 µg/mL	Santa Cruz Biotechnology	sc-8036	https://www.scbt.com/p/his-probe-antibody-h-3

Supplementary Table 1. Antibodies. List of antibodies used in this study including catalogue identifier, working volumes and method of validation.

Supplementary Figures 1

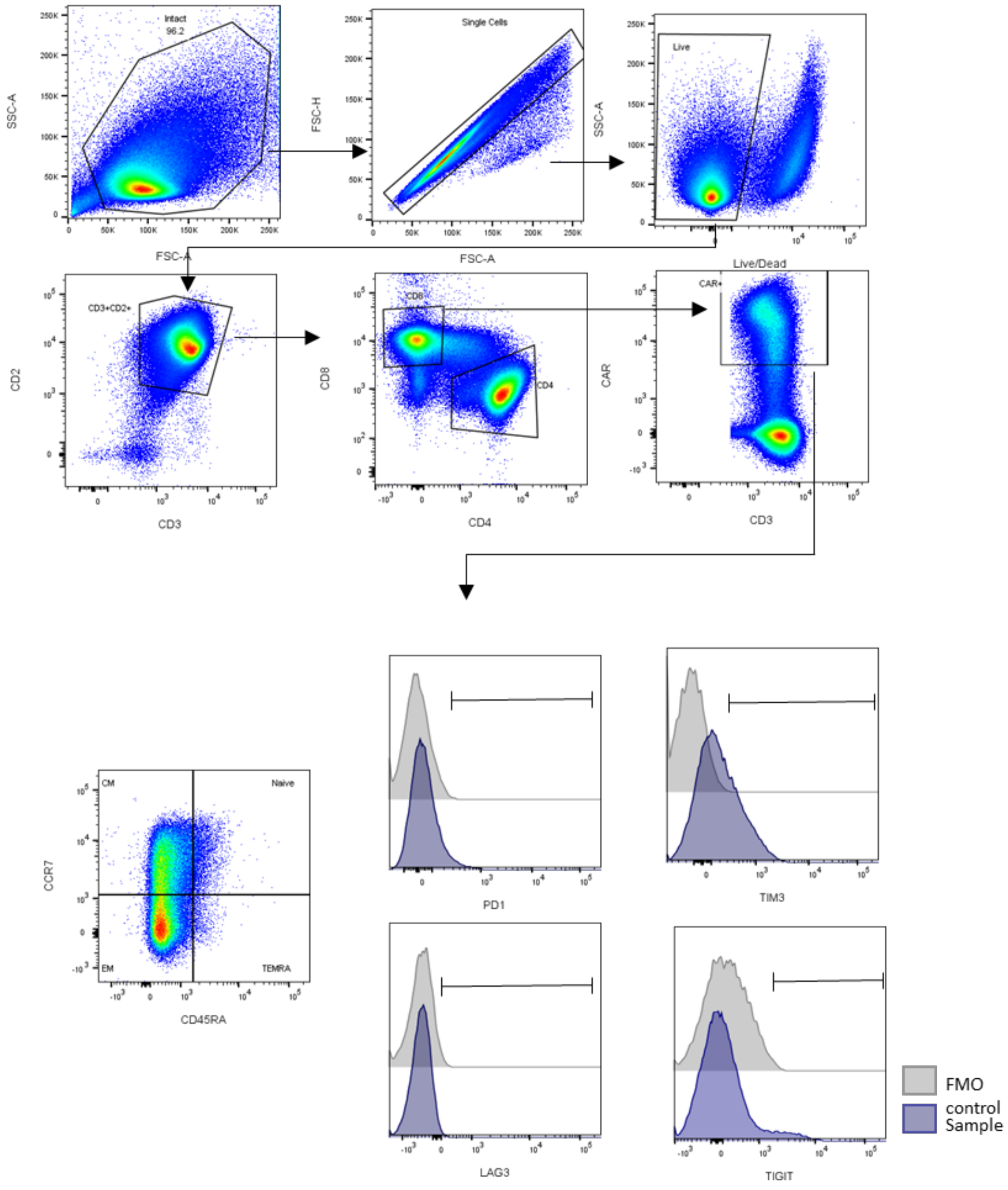

Supplementary Figure 1. Gating strategy for memory and exhaustion phenotypes. Representative example of gating hierarchy employed for the analysis of memory and exhaustion phenotypes in CAR T cell products. Example shows phenotyping of CD8+ CAR T cells; the same analysis was done with CD4+ CAR T cells.

Supplementary Figures 2

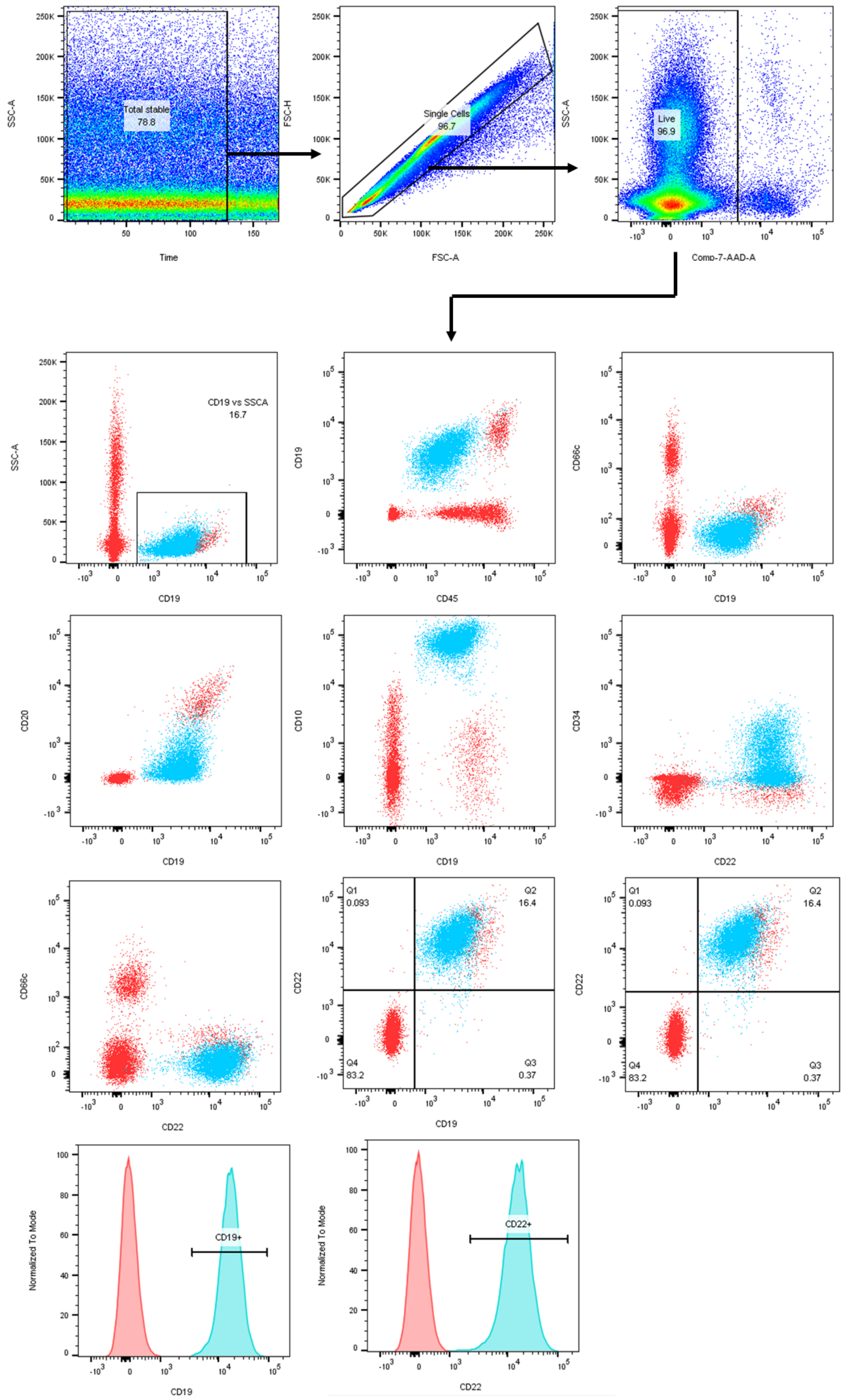

Supplementary Figure 2. Gating strategy for leukemic blasts in bone marrow. Representative example of gating hierarchy employed for the analysis of leukemic blasts in the bone marrow. Blast population is shown in light blue and total live cells are shown in red.
