# Supplementary material for: CAR T cells with dual targeting of CD19 and CD22 in pediatric and young adult patients with relapsed or refractory B cell acute lymphoblastic leukemia: a phase 1 trial
Source: Nat Med. 2021 Oct 12;27(10):1797–805. doi: 10.1038/s41591-021-01497-1 (PMC8516648; doi:10.1038/s41591-021-01497-1)
Supplement: Supplementary file 2 — Reporting Summary [file 41591_2021_1497_MOESM2_ESM.pdf]

Corresponding author(s): Martin Pule

Last updated by author(s): Jul 19, 2021

Reporting Summary

Nature Research wishes to improve the reproducibility of the work that we publish. This form provides structure for consistency and transparency in reporting. For further information on Nature Research policies, see our Editorial Policies and the Editorial Policy Checklist.

Statistics

For all statistical analyses, confirm that the following items are present in the figure legend, table legend, main text, or Methods section.

n/a	Confirmed	
☐	☒	The exact sample size (*n*) for each experimental group/condition, given as a discrete number and unit of measurement
☐	☒	A statement on whether measurements were taken from distinct samples or whether the same sample was measured repeatedly
☐	☒	The statistical test(s) used AND whether they are one- or two-sided<br>*Only common tests should be described solely by name; describe more complex techniques in the Methods section.*
☒	☐	A description of all covariates tested
☒	☐	A description of any assumptions or corrections, such as tests of normality and adjustment for multiple comparisons
☐	☒	A full description of the statistical parameters including central tendency (e.g. means) or other basic estimates (e.g. regression coefficient) AND variation (e.g. standard deviation) or associated estimates of uncertainty (e.g. confidence intervals)
☐	☒	For null hypothesis testing, the test statistic (e.g. *F*, *t*, *r*) with confidence intervals, effect sizes, degrees of freedom and *P* value noted<br>*Give P values as exact values whenever suitable.*
☒	☐	For Bayesian analysis, information on the choice of priors and Markov chain Monte Carlo settings
☒	☐	For hierarchical and complex designs, identification of the appropriate level for tests and full reporting of outcomes
☒	☐	Estimates of effect sizes (e.g. Cohen's *d*, Pearson's *r*), indicating how they were calculated

Our web collection on statistics for biologists contains articles on many of the points above.

Software and code

Policy information about availability of computer code

Data collection	Clinical data are captured in the clinical database via Encapsia ™ electronic data capture (EDC) system v1.0, compliant with the FDA Code of Federal Regulations 21 Part 11 and EU Clinical Trial Directive (EC) No. 2001/20/EC
Data analysis	Clinical data are analyzed using SAS 9.4.<br>Pre-clinical data was analyzed on GraphPad Prism 8, nSolver v2.0.134, R studio 3.6<br>Flow cytometry data was analyzed on FlowJo v10.1.1, FCS Express v7.06.0015 and BD FACSuite v1.3

For manuscripts utilizing custom algorithms or software that are central to the research but not yet described in published literature, software must be made available to editors and reviewers. We strongly encourage code deposition in a community repository (e.g. GitHub). See the Nature Research guidelines for submitting code & software for further information.

Data

Policy information about availability of data

All manuscripts must include a data availability statement. This statement should provide the following information, where applicable:

- Accession codes, unique identifiers, or web links for publicly available datasets
- A list of figures that have associated raw data
- A description of any restrictions on data availability

The raw pre-clinical and clinical datasets generated during and analysed in the current study are not publicly available due to proprietary ownership. All requests for raw and analysed data and materials should be addressed to the corresponding author and will be reviewed by Autolus PLC shortly after to verify whether the request is subject to any intellectual property or confidentiality obligations. Patient data may be subject to patient confidentiality. Any data and materials that can be shared will be released via a material transfer agreement.

Field-specific reporting

Please select the one below that is the best fit for your research. If you are not sure, read the appropriate sections before making your selection.

☒ Life sciences ☐ Behavioural & social sciences ☐ Ecological, evolutionary & environmental sciences

For a reference copy of the document with all sections, see nature.com/documents/nr-reporting-summary-flat.pdf

Life sciences study design

All studies must disclose on these points even when the disclosure is negative.

Sample size	The dose escalation part of the study follows a rolling 6 design (Skolnik et al. 2008). The final sample size is driven by the number of dose cohorts eventually in the study. It was estimated that in the Phase 1 dose escalation up to 24-36 patients in the paediatric / young adult patient cohorts (age 1-24 years) could be enrolled.<br>For the Phase II part of the study (which was not implemented) an original sample size was calculated of up to 24 evaluable paediatric/young adult patients (aged 1-24 years) in total, using a Simon's 2-stage optimal design. This design would have yielded a 1-sided type I error rate of 5% and 80% power when the true response rate was 50%.
Data exclusions	There were no data exclusions.
Replication	Not applicable for clinical data. All pre-clinical replicates were derived from mutually exclusive individual healthy donors. All attempts at replication were successful.
Randomization	The clinical study design is single-arm and non-randomized. Animals in the in vivo pre-clinical experiments were randomly allocated into cohorts.
Blinding	Blinding was not applicable to the clinical study due to the open-label, single arm nature of the study. No blinding protocols were used to generate the pre-clinical data.

Reporting for specific materials, systems and methods

We require information from authors about some types of materials, experimental systems and methods used in many studies. Here, indicate whether each material, system or method listed is relevant to your study. If you are not sure if a list item applies to your research, read the appropriate section before selecting a response.

Materials & experimental systems

n/a	Involved in the study	
☐	☒	Antibodies
☐	☒	Eukaryotic cell lines
☒	☐	Palaeontology and archaeology
☐	☒	Animals and other organisms
☐	☒	Human research participants
☐	☒	Clinical data
☒	☐	Dual use research of concern

Methods

n/a	Involved in the study	
☒	☐	ChIP-seq
☐	☒	Flow cytometry
☒	☐	MRI-based neuroimaging

Antibodies

Antibodies used	The details of the antibodies employed in this study are provided in Supplementary table 1.
Validation	The antibodies used were commercially available and have been validated elsewhere; links to the manufacturer's validation information are provided in Supplementary table 1. The anti-HD37 antibody was manufactured in house and validated as stated in Supplementary table 1. Optimal working dilutions (shown in Supplementary table 1) were selected based on serial dilutions of each individual antibodies and staining index using PBMC.

Eukaryotic cell lines

Policy information about cell lines

Cell line source(s)	All cell lines were sourced from ATCC. These cell lines were SupT1 (CRL-1942), Raji (CCL-86) and Nalm-6 (CRL-3273)
Authentication	Data sheets provided by ATCC upon purchase and staining of cognate markers targeted in this manuscript (CD19 and CD22) verified cell line authenticity. No other varification techniques were used in this study.

Mycoplasma contamination	All cell lines tested negative for mycoplasma using mycoalert mycoplasma detection kit from Lonza
Commonly misidentified lines (See ICLAC register)	No commonly misidentified cell lines were used in the study.

Animals and other organisms

Policy information about studies involving animals; ARRIVE guidelines recommended for reporting animal research

Laboratory animals	All 6-8-week-old female NSG mice were sourced from Charles River. No other animals were used in this study.
Wild animals	No wild animals were used in the study.
Field-collected samples	No field collected samples were used in the study
Ethics oversight	All animal work was performed with the approval of the local Imperial College London Animal Welfare and Ethical Review Body (AWERB) and in compliance with United Kingdom Home Office requirements.

Note that full information on the approval of the study protocol must also be provided in the manuscript.

Human research participants

Policy information about studies involving human research participants

Population characteristics	Male or female paediatric and young adult patients (aged 1-24 years) with high risk (HR) relapsed or refractory B-lineage Acute Lymphoblastic Leukemia. The study patient population was restricted to patients with high risk relapsed or refractory B lineage ALL who are predicted to have a poor outcome even with intensified chemotherapy and stem cell transplant. They must also have had documentation of CD19 and or CD22 expression on leukaemic blasts in the BM, peripheral blood, or cerebrospinal fluid prior to entry.
Recruitment	Patients were recruited from 3 UK pediatric/young adult NHS hospitals (Great Ormond Street Hospital, London; Royal Manchester Children's Hospital; and University College London Hospital). Patients were enrolled based on disease characteristics, suitability and eligibility for the trial. It is not considered there was any selection bias in recruitment of patients into the trial.
Ethics oversight	Study protocol was approved by the following Ethics Committees: NHS Health Research Authority, London -West London &GTAC Research Ethics Committee, The Old Chapel, Royal Standard Place, Nottingham, NG1 6FS, UK

Note that full information on the approval of the study protocol must also be provided in the manuscript.

Clinical data

Policy information about clinical studies

All manuscripts should comply with the ICMJE guidelines for publication of clinical research and a completed CONSORT checklist must be included with all submissions.

Clinical trial registration	ClinicalTrials.gov NCT03289455, EUDRA CT 2016-004680-39.
Study protocol	https://clinicaltrials.gov/ct2/show/results/NCT03289455?term=AUTO3&draw=2&rank=1
Data collection	Three UK pediatric/young adult NHS hospitals were opened for recruitment (Great Ormond Street Hospital, London; Royal Manchester Children's Hospital; and University College London Hospital). The recruitment period ran from July 2017 to September 2019. Data collection ran for 3 years from July 2017 to June 2020.
Outcomes	Primary: Safety; Secondary: Safety and Efficacy. Adverse events were graded according to CTCAE v4.03.<br>CRS was graded according to the Lee criteria (Lee et al. 2018) and neurotoxicity as per the ASBMT guidelines for Immune effector Cell-Associated Neurotoxicity Syndrome (ICANS).<br>Laboratory safety assessments were based on blood samples collected during and after AUTO3 infusion. Samples were analysed locally for complete blood counts, biochemical assays, renal function, hepatic function, coagulation and serum immunoglobulin concentrations<br><br>Response evaluations were based on the response criteria for ALL according to the National Comprehensive Cancer Network guidelines version 2.2014

Flow Cytometry

Plots

Confirm that:

☒ The axis labels state the marker and fluorochrome used (e.g. CD4-FITC).

☒ The axis scales are clearly visible. Include numbers along axes only for bottom left plot of group (a 'group' is an analysis of identical markers).

☒ All plots are contour plots with outliers or pseudocolor plots.

☒ A numerical value for number of cells or percentage (with statistics) is provided.

Methodology

Sample preparation	PBMC, leukapheresates and cell products were used fresh or after cryopreservation. Cells were first stained with fixable viability dye diluted in PBS for 10 minutes. For staining of CCR7, cells were incubated with anti-CCR7 at 37C for 30 min. This was followed by surface staining with a master mix of antibodies diluted in BD brilliant stain buffer (BD, 563794) for 30 min. For intracellular stain, the cells were fixed and permeabilized using the Foxp3/transcription factor staining buffer set (ThermoFisher, 00-5523-00) as per manufacturer's instructions.
Instrument	Pre-clinical work: Miltenyi MACSQuant X; Clinical samples : BD FACSLyric Clinical System and BD LSRFortessa X20
Software	Bone marrow flow cytometry data were analyzed using FlowJo v10.6.1. Analysis of immunophenotype of drug products and leukapheresis were analyzed using FCS Express v7.06.0015 for template analysis. Analysis of peripheral blood clinical samples was done using BD FACSuite v1.3.
Cell population abundance	Cell sorting was not employed in this study.
Gating strategy	All cells were gated on FSC-A vs SSC-A morphology, stable acquisition over time, doublet exclusion on FSC-H vs FSC-A and live cells (negative staining for fixable viability dye) as shown in supplementary figures 1-2. Downstream gating is described for each particular experiment.

☒ Tick this box to confirm that a figure exemplifying the gating strategy is provided in the Supplementary Information.
